# Distinct sFOLR1 and Cytokine Secretion Profiles Across Marker-Enriched OVCAR-3 Ovarian Cancer Cell Subpopulations: A Pilot Study

**DOI:** 10.3390/biom16101462

**Published:** 2026-10-07

**Authors:** Andrei Pașca, Eva Fischer-Fodor, Paul Milan Kubelac, Ovidiu Bălăcescu, Oana Baldasici, Bogdan Pop, Natalia Miklášová, Nicoleta-Monica Pașca, Cătălin Ioan Vlad, Patriciu Andrei Achimaș-Cadariu

**Affiliations:** 1 Department of Surgical Oncology and Gynecologic Oncology, ‘Iuliu Hațieganu’ University of Medicine and Pharmacy, 400012 Cluj-Napoca, Romania; 2 Department of Surgical Oncology, “Prof. Dr. Ion Chiricuta” Institute of Oncology, 400015 Cluj-Napoca, Romania; 3 Tumor Biology Department, “Prof. Dr. Ion Chiricuta” Institute of Oncology, 400015 Cluj-Napoca, Romania; 4 Department of Medical Oncology, “Prof. Dr. Ion Chiricuta” Institute of Oncology, 400015 Cluj-Napoca, Romania; 5 Department of Functional Genomics, Proteomics and Experimental Pathology, “Prof. Dr. Ion Chiricuta” Institute of Oncology, 400015 Cluj-Napoca, Romania; 6 Department of Pathology, “Prof. Dr. Ion Chiricuta” Institute of Oncology, 400015 Cluj-Napoca, Romania; 7 Department of Inorganic Chemistry, Faculty of Natural Sciences, Comenius University Bratislava, Ilkovičova 6, Mlynská dolina, 84215 Bratislava, Slovakia; natalia.miklasova@uniba.sk

**Keywords:** ovarian cancer, OVCAR-3, soluble FOLR1, folate receptor alpha, marker-enriched fractions, CD133, EpCAM, CD90, ABCG2, cytokines

## Abstract

Background: Folate Receptor Alpha (FRα), encoded by the Folate Receptor 1 (FOLR1) gene, is expressed in many high-grade serous ovarian carcinomas, but the tumor-cell phenotypes contributing to soluble FOLR1 (sFOLR1) release remain unclear. This pilot study compared sFOLR1 and cytokine secretion across OVCAR-3 fractions enriched for CD133, EpCAM/CD326, CD90, and ABCG2/CD338. Methods: The parental OVCAR-3 culture was phenotyped by flow cytometry and separated into marker-positive and marker-negative fractions using CD133, EpCAM/CD326, and ABCG2/CD338 immunomagnetic selection. CD90-positive and corresponding negative fractions were isolated after indirect co-culture of OVCAR-3 cells with primary mesenchymal stromal cells. Paired fractions were cultured for 120 h, and sFOLR1, vascular endothelial growth factor (VEGF), interleukin (IL)-6, and IL-10 were measured at 24 h intervals. Results: sFOLR1 release varied markedly by time and phenotype. CD133-positive and CD326-positive fractions showed time-dependent increases; CD90-positive maintained low, comparatively stable concentrations; and CD338-positive declined from a high early value, while the corresponding negative fraction increased. In CD133-positive and CD326-positive fractions, sFOLR1 tracked with VEGF and/or IL-6, whereas CD90-positive and CD338-positive fractions showed distinct, partly uncoupled cytokine patterns. Conclusions: These exploratory associations in a single cell line support further investigation of sFOLR1 and cytokine profiles.

## 1. Introduction

Ovarian malignancies account for a significant portion of cancer-related mortality among women. According to GLOBOCAN, 330,731 new cases were registered worldwide in 2024, with 203,850 deaths [1]. Little progress has been made in diagnostic efforts using biomarkers [2,3], while no screening strategies have proven efficient across long-term assessment [4]. Therefore, most cases are currently diagnosed at advanced stages where standard therapeutic options usually involve a combination of platinum-based chemotherapy and cytoreductive surgery adjusted to clinical and molecular features [5]. Although most cases exhibit platinum sensitivity, with initial clinical responses, the majority of patients will relapse, signifying both exceptions related to cell-intrinsic resistance or microenvironment-dependent adaptation [6]. Even when recent advances in therapeutic options regarding ovarian cancer have proven the efficacy of various treatments such as antiangiogenic agents, poly ADP-ribose polymerase inhibitors (PARPis) or immunotherapy [7,8,9], a platinum-resistant disease truly remains a challenge.

Miscellaneous biomarkers have been investigated as diagnostic tools, such as serum Cancer Antigen 125 (CA-125). Nevertheless, limited specificity and sensitivity prompted investigation of complementary parameters, including human epididymis protein 4, mesothelin, and cell-free DNA fragmentation profiles [10], various diagnostic scores [11], and alternative markers such as cytokines [12]. Other markers were studied to highlight molecular signatures and support risk stratification, treatment selection, or disease monitoring. Folate Receptor alpha (FRα), encoded by FOLR1, is a glycosylphosphatidylinositol-anchored folate-binding protein overexpressed in several epithelial malignancies, including ovarian carcinoma. Soluble FOLR1 (sFOLR1) exhibits extracellular FRα protein derived from the FOLR1 gene and can be measured in serum or ascites. Release of membrane-associated FRα may involve cleavage of its glycosylphosphatidylinositol anchor or proteolytic shedding [13]. Higher values of sFOLR1 were identified in patients with ovarian cancer [14] and have been associated with disease stage, tumor grade, progression-free survival, and treatment response [15]. Moreover, recent publications have identified FOLR1 as a therapeutically actionable antigen, enabling the introduction of the antibody-drug conjugate mirvetuximab–soravtansine, currently in advanced clinical trials [16,17], and the development of FOLR1-targeting CAR-T cells for immunotherapy [18].

Despite extensive characterization of FOLR1 expression, its origin and biological context remain pending. Different histologies have various patterns of FOLR1 expression [19]. Moreover, discordant sFOLR1 and CA-125 kinetics in recurrent disease suggest that cell populations producing sFOLR1 may be biologically different from those secreting CA-125. Therefore, recognizing distinct phenotypes expressing sFOLR1 is paramount for precisely interpreting values and for comprehending its relationship to chemoresistant disease.

Further functional evaluation of tumor cells can be studied through various cytokines such as IL-6 (promoted ovarian cancer cell growth, invasion, stemness, and platinum resistance [20,21]), IL-10 (proven roles in tumor proliferation, migration, apoptosis resistance, and immune evasion [22]) or VEGF (proven angiogenic signaling role, frequently associated with aggressive disease). Cancer stem-like cells contribute to dissemination, relapse, and treatment resistance [23]. Relevant phenotypes include EpCAM/CD326-positive epithelial cells, CD44-positive cells, aldehyde dehydrogenase-high cells, Prominin-1/CD133-positive cells, and Thy-1/CD90-positive mesenchymal-like cells, which have emerged as hallmarks of these behaviors [24,25,26,27]. CD90 has been previously linked to self-renewal, migration, and chemoresistance [28,29,30]. The ATP-binding cassette transporter ABCG2/CD338 mediates drug efflux and was associated with a treatment-resistant cell line [31,32]. These cell subpopulations partly overlap but are non-equivalent phenotypes that may differ in secretory behavior.

Consequently, this pilot investigation sought to compare time-dependent extracellular sFOLR1 concentrations and their association with VEGF, IL-6, and IL-10 cytokines measured from EpCAM/CD326-positive, CD133-positive, CD90-positive, and ABCG2/CD338-positive OVCAR-3 cell fractions and their corresponding marker-negative populations. This study hypothesized that sFOLR1 secretion would vary by cellular phenotype and that distinct associated cytokine secretion profiles could provide additional descriptive information about OVCAR-3 subpopulations.

## 2. Materials and Methods

### 2.1. Cell Line and Culture Conditions

The human OVCAR-3 (NIH: OVCAR-3) ovarian carcinoma cell line, product number 300307 was purchased from CLS GmbH (via Cytion, Eppelheim, Germany). OVCAR-3 was established from malignant ascites of a female patient and has the molecular features of a high-grade serous ovarian carcinoma [33]. Cells were maintained in RPMI-1640 medium containing 2.0 mM glutamine and 2.0 g/L NaHCO_3_ and supplemented with 20% fetal bovine serum and 0.01 mg/mL insulin (Capricorn Scientific, Ebsdorfergrund, Germany). Cells were then subculted and propagated in 75 cm^2^ Nunclon Delta treated flasks (Thermo Fisher Scientific, Waltham, MA, USA), detached at 80–85% subconfluence using Accutase (Capricorn Scientific), centrifuged, and plated for experiments on multiwell assay plates at 1 × 10^5^ cells/mL concentrations. All ovarian cancer fractions investigated in this study were derived from the same parental OVCAR-3 line.

### 2.2. Flow-Cytometric Characterization

OVCAR-3 cells were harvested, assessed for viability with Trypan Blue (Sigma-Aldrich, St. Louis, MO, USA), and counted using an EVE automated cell counter (NanoEnTek, Seoul, Republic of Korea). After treatment with Fc-blocking reagent (Miltenyi Biotec, Bergisch Gladbach, Germany), cells were resuspended in bovine serum albumin-containing staining buffer (BD Pharmingen, BD Biosciences, San Jose, CA, USA) to a density of 1 × 10^6^ cells/mL. Four batches were individualized, and aliquots of 100 µL were dispensed into 6 mL test tubes; cell-surface staining was performed with phycoerythrin (PE)-conjugated anti-FOLR1, PE-conjugated anti-CD133/Prominin-1, PE-conjugated anti-ABCG2/CD338, or PerCP/Cyanine5.5-conjugated anti-CD90/Thy-1 antibodies (BioLegend, San Diego, CA, USA) at the manufacturer’s recommended concentrations. Cells were washed with buffer, resuspended in 500 µL BD Cytofix buffer, labelled, and passed through 70 µm syringe-type sterile filters. Finally, the cells were subjected to flow-cytometric evaluation using an S3e Cell Sorter (Bio-Rad, Hercules, CA, USA). Flow cytometry characterized cell-surface markers in the parental OVCAR-3 culture before immunomagnetic enrichment. ELISA subsequently measured sFOLR1 in the culture supernatants of each fraction at 24, 48, 72, 96 and 120 h. Post-isolation purity and parallel measurement of cell-surface FOLR1 in the individual fractions were not assessed.

### 2.3. Immunomagnetic Isolation of OVCAR-3 Cell Subpopulations

Cells from four to five 75 cm^2^ flasks at 75–80% confluence were enzymatically detached, centrifuged, washed with phosphate-buffered saline (PBS), and divided into 3 batches of 1.5 × 10^7^ cells. The 90 μL separation buffer consisted of PBS with 0.05% bovine serum albumin and 2 mM EDTA and was passed through 0.22 µm Millex filters (Merck, Darmstadt, Germany). Each batch underwent single-step positive selection on magnetic columns (MS or LS) using MiniMacs and MidiMACS separators (magnetic separation columns and devices from Miltenyi Biotec, Bergisch Gladbach, Germany). We used human CD133, CD326/EpCAM, and ABCG2/CD338 MicroBead kits (Miltenyi Biotec, Auburn, CA, USA). For positive enrichment of the CD133 population, 30 μL FcR Blocking reagent was added to the OVCAR-3 cell suspension, along with 30 μL of anti-CD133 magnetic beads, and the suspension was gently mixed and incubated for 15 min at 2–8 °C. The cells were washed twice in 2 mL cold buffer, counted, and resuspended in cold 500 μL buffer. The separation columns were placed in the magnetic field of the MACS separator, washed with buffer, and then the cell suspension was loaded onto the columns under the magnetic field. The unlabelled, CD133-negative cells flowed through the columns, and they were collected in a test tube. The positively selected, labelled cells were magnetically retained on the columns. After removing the columns from the magnetic field, 1 mL buffer was dispensed onto the columns, and the cells were eluted by pumping with the syringe piston of the separation device. An average of 11.4% CD133+ cells were collected; both the CD133-positive enriched fraction and the CD133− cell populations were washed and resuspended in cell culture media. A similar procedure was used to perform ABCG2/CD338 enrichment of monoculture OVCAR-3 cells; the recovery of the CD338-enriched fraction was 55.9%. ABCG2/CD338 has been discussed in relation to chemoresistance in previous work [34]. Instead, for EpCAM/CD326, a different stoichiometry was used, according to the manufacturer’s indications: to the same amount of 1.5 × 10^7^ cells, a total volume of 300 μL buffer was added, 100 μL FcR-Blocking reagent, and 100 µL of CD326 (EpCAM) MicroBeads were incubated with the cells for 30 min in the refrigerator. The remaining steps were performed identically to the other two cell surface markers, and yielding a median recovery of 43.2% for the CD326+ cells. The whole separation process was repeated 3 times for all targets, with OVCAR-3 cells from three successive passages. These percentages describe recovery by immunomagnetic separation, not post-isolation marker purity.

### 2.4. Enrichment of CD90-Positive Cells After Indirect Co-Culture with Mesenchymal Stromal Cells

Because CD90-positive cells were infrequent in the parental OVCAR-3 culture, we adapted an indirect co-culture protocol from Yang et al. [35]. OVCAR-3 cells were dispensed in six-well cell culture plates at 1 × 10^5^ cells/mL in 2.5 mL of supplemented RPMI-1640 medium. Primary human umbilical cord-derived mesenchymal stromal cells product number 500-010 (ATCC; supplied by LGC Standards, Wesel, Germany) were cultivated in Mesenchymal Stem Cell Growth Medium 2 (PromoCell, Heidelberg, Germany) supplemented with 5% fetal bovine serum, 1% penicillin–streptomycin, 2 ng/mL recombinant human fibroblast growth factor, and 2 ng/mL epidermal growth factor (Sigma-Aldrich). Permeable membrane inserts (0.4 μm pore size, Corning Transwell, Corning, Kennebunk, ME, USA; from Merck, Darmstadt, Germany) were positioned in each well of the tumor-cell-loaded 6-well plates. Inside these inserts, mesenchymal stromal cells were seeded at 5 × 10^4^ cells/mL in a total volume of 500 µL medium. After 72 h of incubation, the inserts were removed, and OVCAR-3 cells were harvested from the bottom by trypsinization (Trypsin-EDTA solution, Sigma-Aldrich), washed, and magnetically separated. For CD90+ enrichment, the human anti-CD90/Thy-1 microbead kit was used (Miltenyi Biotec); 10^7^ cells were suspended in 80 μL buffer, mixed with 20 μL anti-CD90 magnetic microbeads, and kept for 15 min in a refrigerator. After two washing steps with 2 mL of cold buffer, the cells were suspended in 500 μL of buffer and subjected to magnetic separation as above. The median yield of the separation procedure was 7.9%. In the parental monoculture, CD90+ events accounted for 2.58% of the displayed gated population (Figure S1f). Magnetic recovery and flow-cytometric marker frequency are different measurements and do not quantify the same endpoint. The 0.4 μm membrane physically separated the stromal cells in the inserts from the cells in the lower compartment, and the inserts were removed before the lower-compartment cells were harvested. This design limits direct cell mixing but does not independently verify the identity or purity of the recovered cells. Both CD90-positive and CD90-negative fractions had undergone the same preceding co-culture. The primary mesenchymal stromal cells were a separate component of the indirect co-culture system, not an additional ovarian cancer cell line. Both recovered CD90-defined fractions were derived from the OVCAR-3 cultures in the lower compartment, with identity and purity limitations as described above.

### 2.5. Sample Collection

Marker-positive (CD133+, CD326+, CD338+, and CD90+) and corresponding marker-negative fractions were seeded in 24-well Nunclon plates at 1 × 10^5^ cells/mL in 1.0 mL medium. We prepared five wells for each fraction and included cell medium as a reference. Supernatants were collected after 0 (blank), 24, 48, 72, 96, and 120 h, centrifuged at 10,000 rpm, aliquoted, and conserved at −80 °C. The pellets were collected, resuspended in PBS and subjected to automated Trypan Blue cell counting using an EVE NanoEnTek bench counter (NanoEnTek Inc., Seoul, Republic of Korea). The total protein content of the supernatants was measured using Bradford reagent (Bio-Rad) against 1.56–100 µg/mL bovine serum albumin standards (Capricorn Scientific). Absorbance was read at 590 nm using a Synergy LX multimode reader (BioTek Instruments, Inc., Winooski, VT, USA) with Gen5 software version 2.0 (BioTek Instruments, Winooski, VT, USA). Samples were collected in triplicate from three distinct assay plates. The sampling focused on conditioned-medium profiles after an initial 24 h culture interval, allowing extracellular analytes to accumulate before the first collection. Within-fraction comparisons used the first measurement at 24 h as the reference.

### 2.6. Time-Dependent Metabolic Activity of OVCAR-3 Subsets

Time-dependent metabolic activity in the OVCAR-3 cell subset was evaluated over 120 h using an Alamar Blue resazurin-based dye solution (Invitrogen, Thermo Fisher Scientific, Waltham, MA, USA) as per published protocols [36]. After medium collection, the wells were washed with 0.5 mL PBS and incubated for 4 h with 400 µL of 10% Alamar Blue in RPMI-1640 containing 10% fetal bovine serum. Fluorescence was measured at 540/620 nm using the Synergy LX reader after subtraction of cell-free and reagent background values. Fluorescence intensity was used as an indicator of viable-cell metabolic activity. Fluorescence data were analyzed using the GraphPad Prism 5 software (GraphPad Software, La Jolla, CA, USA). This assay measured metabolic activity during culture without a drug challenge; it did not validate sensitivity to chemotherapy or the self-renewal and tumor-initiating properties of the selected fractions.

### 2.7. Quantification of sFOLR1 and Cytokines

sFOLR1 was measured using the Human FOLR1 DuoSet ELISA kit and DuoSet ELISA Ancillary Reagent Kit 2 (R&D Systems, Minneapolis, MN, USA) as described earlier [37]. The Duo-Set was supplemented with 3 insert-type 96-well assay plates, buffers, TMB substrate, wash buffer and other additional materials of the DuoSet ELISA Ancillary Reagent Kit 2, provided by the same company. We evaluated 8 standards for sFOLR1 in duplicate for each assay plate. On the three plates, control samples were distributed in triplicate: 40 fraction-time combinations of the CD133+/−; CD326+/−; CD90+/−; and CD338+/− fractions (8 fractions × 5 collection times: 24, 48, 72, 96, and 120 h after plating), before replication, color controls and blank values consisting of samples harvested at 0 h, immediately after the cells’ resuspension in culture media. The 0 h blank values reflected the sFOLR1 and cytokine levels in the cell culture media before the cells began secreting these target proteins. Collection times were 24, 48, 72, 96, and 120 h after plating; the 40 fraction–time combinations represent sampling conditions within the parental OVCAR-3 model. All wash steps were performed with an automated washer (DiaSource Diagnostics, Louvain-la-Neuve, Belgium), the benches were measured with the Synergy LX reader, and the concentrations were provided by the Gen5 software version 2.0 incorporated in the system. The concentrations were expressed as a fraction of the target protein (e.g., sFOLR1) and the total protein content, and normalized up to 10^6^ counted cells, as the number of cells was substantially different after 96 or 120 h due to cell proliferation, especially in the negative cellular subpopulations. Data were analyzed using GraphPad Prism 5 (GraphPad software, La Jolla, CA, USA) and Statistica 12.5 (StatSoft Inc., Tulsa, OK, USA). In this study, sFOLR1 denotes extracellular FOLR1 detected by ELISA in the collected supernatants. This ELISA does not distinguish changes in cellular receptor abundance from changes in receptor release, nor does it identify the molecular release mechanism.

To quantify vascular endothelial growth factor A (VEGF-A), a Human VEGF PicoKine ELISA Kit was used (BosterBio, Pleasanton, CA, USA), following the manufacturer’s protocol [38]. Interleukin-6 (IL-6) was assessed using the Human IL-6 Enzyme-linked immunosorbent assay kit (Elabscience Biotechnology Inc., Houston, TX, USA). R&D Systems provided the assay kits for interleukin-10 (Human IL-10 Duo-set ELISA kit). Three assay kits were used for each cytokine, with three coated assay plates and the related detection and capture antibodies, recombinant standard proteins, buffers, diluents, TMB substrate, horseradish peroxidase (HRP) solution, and wash solutions. The equivalent samples and controls were measured as in sFOLR1 detection (triplicates).

### 2.8. Data Processing and Statistical Analysis

Statistical analysis was performed using GraphPad Prism 5 (GraphPad Software, La Jolla, CA, USA) and Statistica 12.5 (StatSoft, Tulsa, OK, USA). The experimental design comprised 8 fractions arranged as four marker-positive/marker-negative pairs sampled at five time points (24, 48, 72, 96, and 120 h). The reported analyte values are expressed as pg per mg of total protein measured in the corresponding supernatant (pg/mg protein). Five culture wells were prepared per fraction, and supernatants were collected in triplicate from the three assay plates. Immunomagnetic isolation was repeated using cells from the three successive passages.

Within-fraction temporal comparison used the 24 h measurement as the reference. The differences across cell subsets were evaluated using one-way repeated-measures analysis of variance (ANOVA) for comparisons across the sampling times and cell fractions, and two-way repeated-measures ANOVA within each marker-defined enriched/depleted pair, with time and marker status as factors. Bonferroni post-tests compared the 48, 72, 96 and 120 h measurements with the 24 h reference.

Associations between sFOLR1, VEGF, IL-6, and IL-10 were explored using Pearson and Spearman coefficients of correlation; principal component analysis (PCA) was deployed to explore covariance among the four analytes with component contributions reported as percentages of explained variance. We used distance-weighted least-squares surface and contour plots for exploratory visualization. The experimental work involved three successive isolation passages and triplicate sampling from the three assay plates (Section 2.3 and Section 2.5); the five sampling times do not represent five independent biological replicates. Correlation, PCA, and fitted surfaces are exploratory descriptions of the time-dependent profiles. Shared temporal trends can contribute to apparent associations, and PCA loading proximity or fitted surface geometry does not establish a statistically significant or causal relationship.

## 3. Results

### 3.1. Baseline Phenotype and Recovery of OVCAR-3 Subpopulations

Flow cytometry identified an FOLR1-rich parental OVCAR-3 population. Within the displayed gated population, the measured positive fraction was 81.29% for membrane FOLR1, detected by PE-labelled anti-FR-α antibody. Other expressions were 7.59% for CD133, 92.70% for ABCG2/CD338, and 2.58% for CD90; detailed measurements are provided in the Supplementary Material (Figure S1a–f).

The OVCAR-3-derived marker-defined fractions showed different morphologies under microscopy (Supplementary Figure S2a–d) and different time-dependent resazurin fluorescence profiles (Supplementary Figure S3).

### 3.2. Phenotype- and Time-Dependent Secretion of sFOLR1, VEGF, IL-6, and IL-10

**Figure 1:**
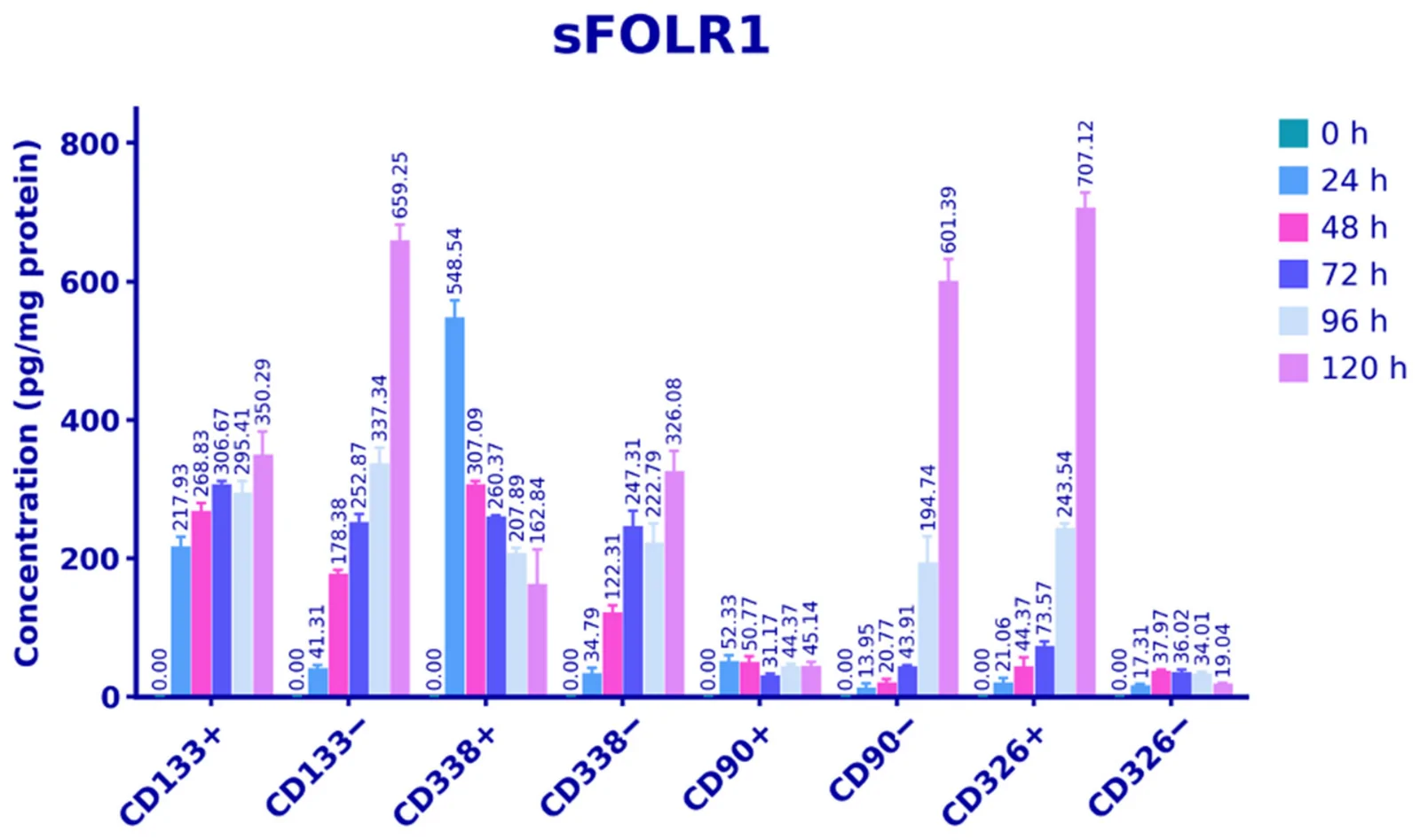
Soluble FOLR1 concentration in marker-enriched and corresponding depleted OVCAR-3 fractions. Bars show means ± standard error of the mean (SEM); + and − indicate marker-positive and marker-negative fractions, respectively; 0 h denotes the medium blank.

The eight OVCAR-3 cell subpopulations displayed markedly different sFOLR1 trajectories between 24 and 120 h after plating. Figure 1 depicts time-dependent variation in extracellular sFOLR1 concentration, measured against the total supernatant protein content of the samples and normalized to 10^6^ counted cells. Across the eight cell fractions, one-way repeated-measures ANOVA indicated an overall effect of sampling time on sFOLR1 concentration at 24 h and at 120 h, and between 48 h and 120 h of incubation, regardless of the cell subset (Bonferroni post-test, F = 3.389, r2 = 0.3262, *p* = 0.0221).

Within each marker-positive and marker-negative pair, temporal comparisons were reported using two-way repeated-measures ANOVA with Bonferroni post-tests. Relative to 24 h, sFOLR1 increased in CD133-positive fractions at 72, 96, and 120 h and in CD133-negative fractions at each subsequent time frame. CD338-positive fractions showed the opposite pattern, with a progressive decline from a high 24 h concentration, whereas CD338-negative fractions increased at 72, 96, and 120 h. CD90-positive fractions remained low and comparatively stable, while CD90-negative fractions increased sharply at 96 and 120 h. CD326-positive fractions increased at 72, 96, and 120 h, whereas CD326-negative fractions remained low. Detailed measurements are available in the Supplementary Material, Figure S4a–d.

**Figure 2:**
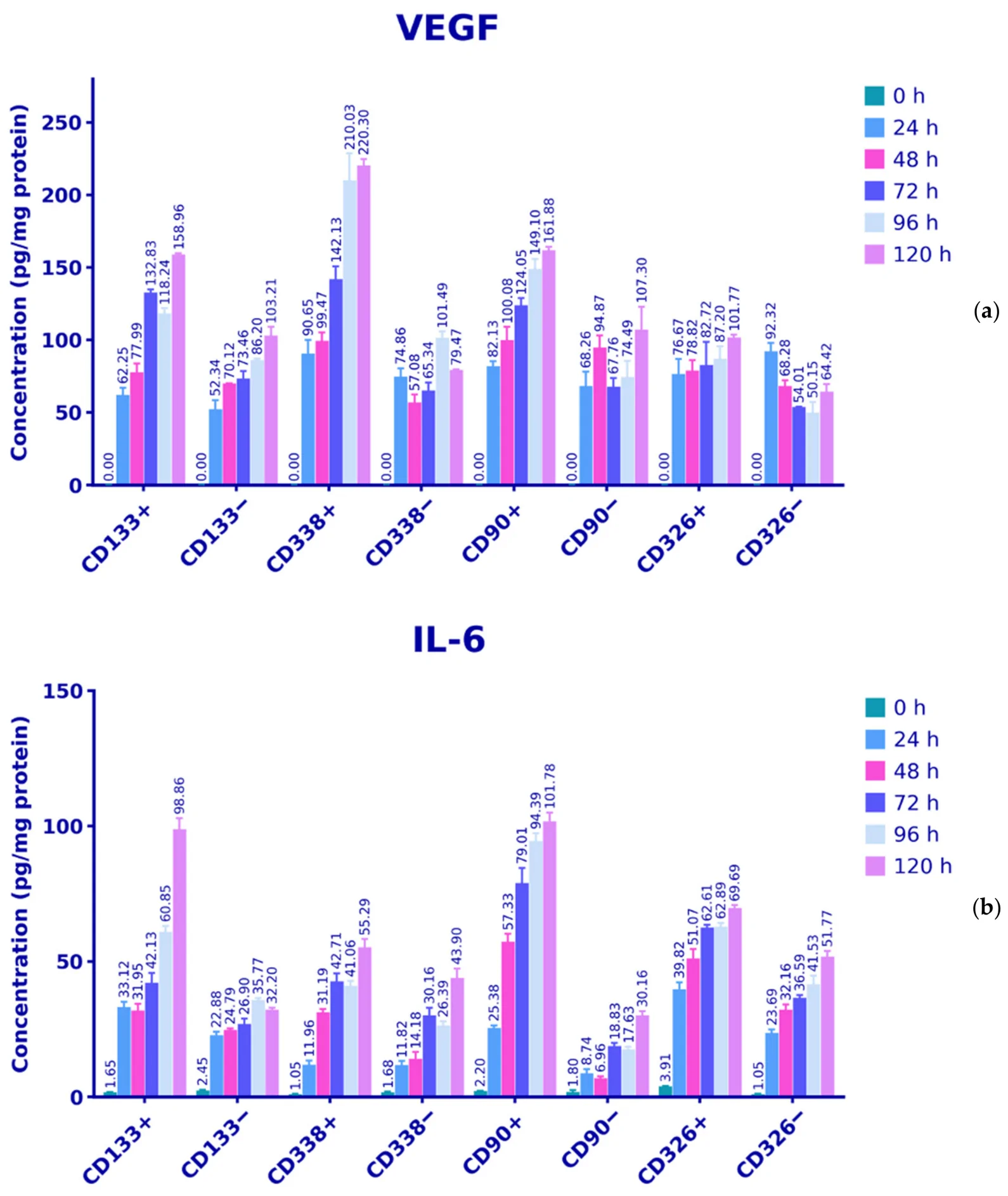

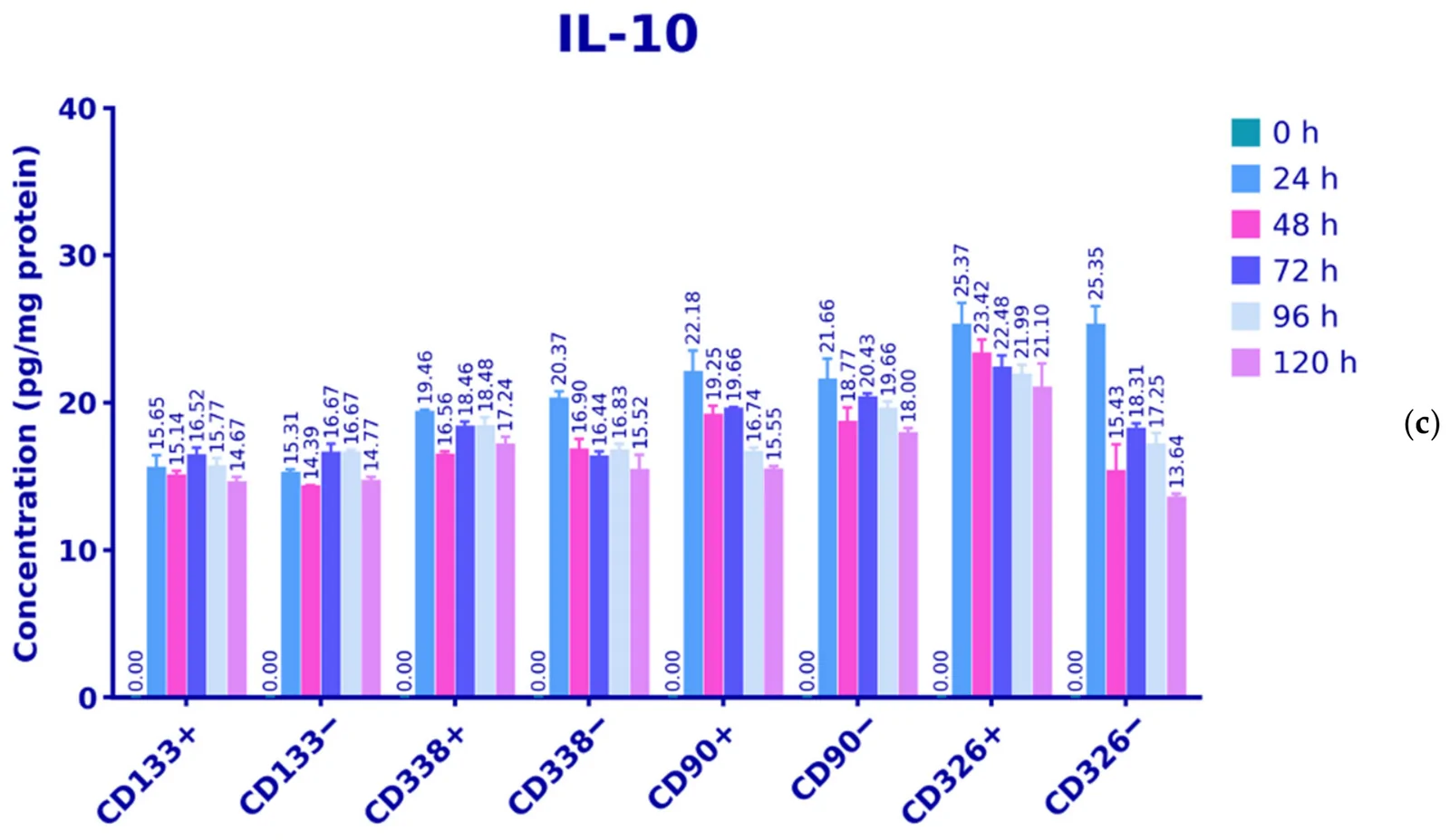
Concentrations of (**a**) vascular endothelial growth factor (VEGF), (**b**) interleukin-6 (IL-6), and (**c**) interleukin-10 (IL-10) in marker-enriched and corresponding depleted OVCAR-3 fractions. Bars show means ± standard error of the mean (SEM); + and − indicate marker-positive and marker-negative fractions, respectively; 0 h denotes medium blank.

In fractions enriched for EpCAM/CD326, Thy-1/CD90, Prominin-1/CD133, and ABCG2/CD338+, the extracellular IL-6, IL-10, and VEGF concentrations showed distinct temporal patterns among the OVCAR-3 fractions (Figure 2a–c). They exhibited phenotype-dependent kinetics. Relative to 24 h, VEGF increased at 72, 96 and 120 h in the CD133-positive, CD338-positive, and CD90-positive fractions, but decreased in CD326-negative fractions at 72 and 96 h (Table S1). IL-6 increased at 48, 72, 96 and 120 h in CD338+, CD326+, and CD90+ fractions; at 96 and 120 h in CD133+ fractions; at 96 h in CD133-negative fractions; at 72, 96, and 120 h in CD338-negative and CD326-negative fractions; and at 72 and 120 h in CD90-negative fractions (Table S2).

IL-10 predominantly decreased (Table S3). Lower concentrations were reported at 48, 72, 96, and 120 h in CD338-negative, CD326-negative, and CD90-positive fractions; at 72, 96, and 120 h in CD326-positive fractions; at 48 h in CD338-positive fractions; and at 48 and 120 h in CD90-negative fractions.

### 3.3. CD133-Defined Fractions: Distinctive Pattern of FOLR1/IL-10/IL-6/VEGF Levels in CD133-Positive and CD133-Negative Fractions

**Figure 3:**
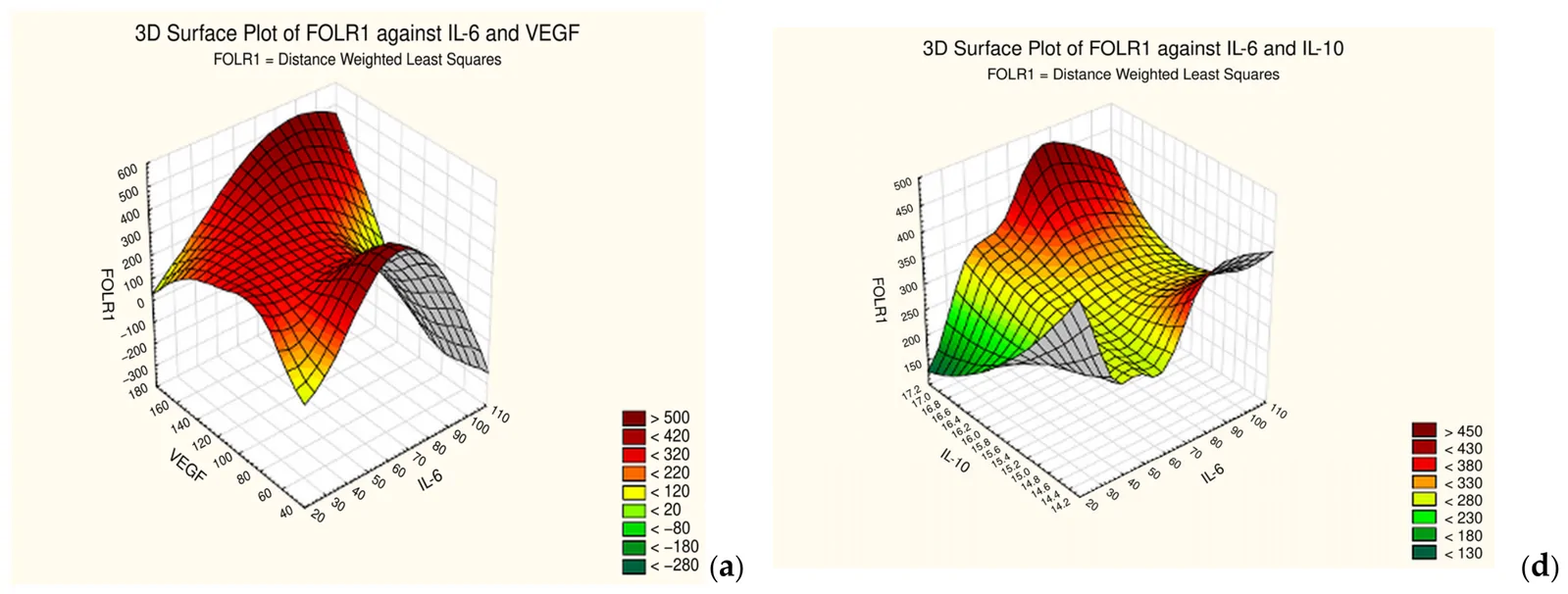

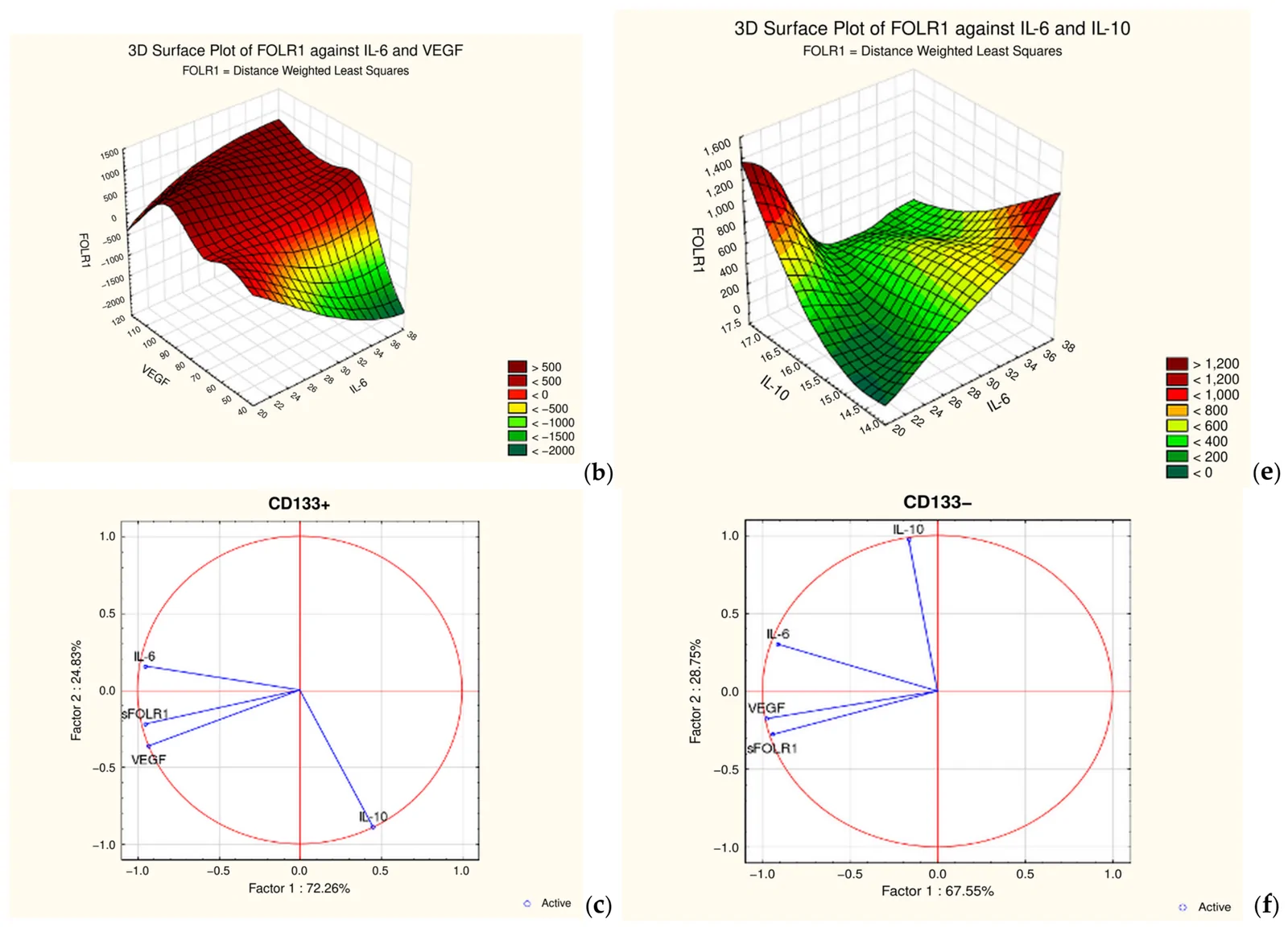
Exploratory relationships among sFOLR1, IL-6, IL-10, and VEGF in CD133-defined OVCAR-3 fractions over 120 h. (**a**,**d**) Distance-weighted least-squares surfaces for CD133-positive fractions; (**b**,**e**) corresponding surfaces for CD133-negative fractions; (**c**,**f**) principal component loading plots for the two fractions. Surface regions outside the observed measurement range represent model extrapolation and should not be interpreted as experimental values.

In CD133-positive fractions, higher sFOLR1 values coincided with higher VEGF and IL-6 concentrations (Figure 3a,d). The first PCA component accounted for 72.26% of the variance (Figure 3c). sFOLR1 correlated with VEGF (Spearman r = 1.000, one-tailed *p* = 0.0083) and with IL-6 (Pearson r = 0.8254, one-sided *p* = 0.0426), but no association was identified with IL-10. The sFOLR1 temporal profiles in CD133-positive and CD133-negative fractions were positively associated (Pearson r = 0.9331, one-tailed *p* = 0.0103). Within the CD133-negative fraction, sFOLR1 was positively associated with VEGF and with IL-6 concentrations (Spearman r = 0.900, one-tailed *p* = 0.0417). Within the CD133-negative fractions, sFOLR1 was not associated with IL-10 (Pearson r = −0.0888, one-tailed *p* = 0.4436). In the CD133-negative fractions, the modelled relationship with IL-10 extended to sFOLR1 concentrations above 1200 pg/mg protein, outside the experimentally observed range (Figure 3e), and did not establish an association with the measured data.

### 3.4. EpCAM/CD326-Defined Fractions: Distinctive Pattern of sFOLR1 and Cytokines in EpCAM/CD326+ Versus CD326− Subsets from OVCAR-3

**Figure 4:**
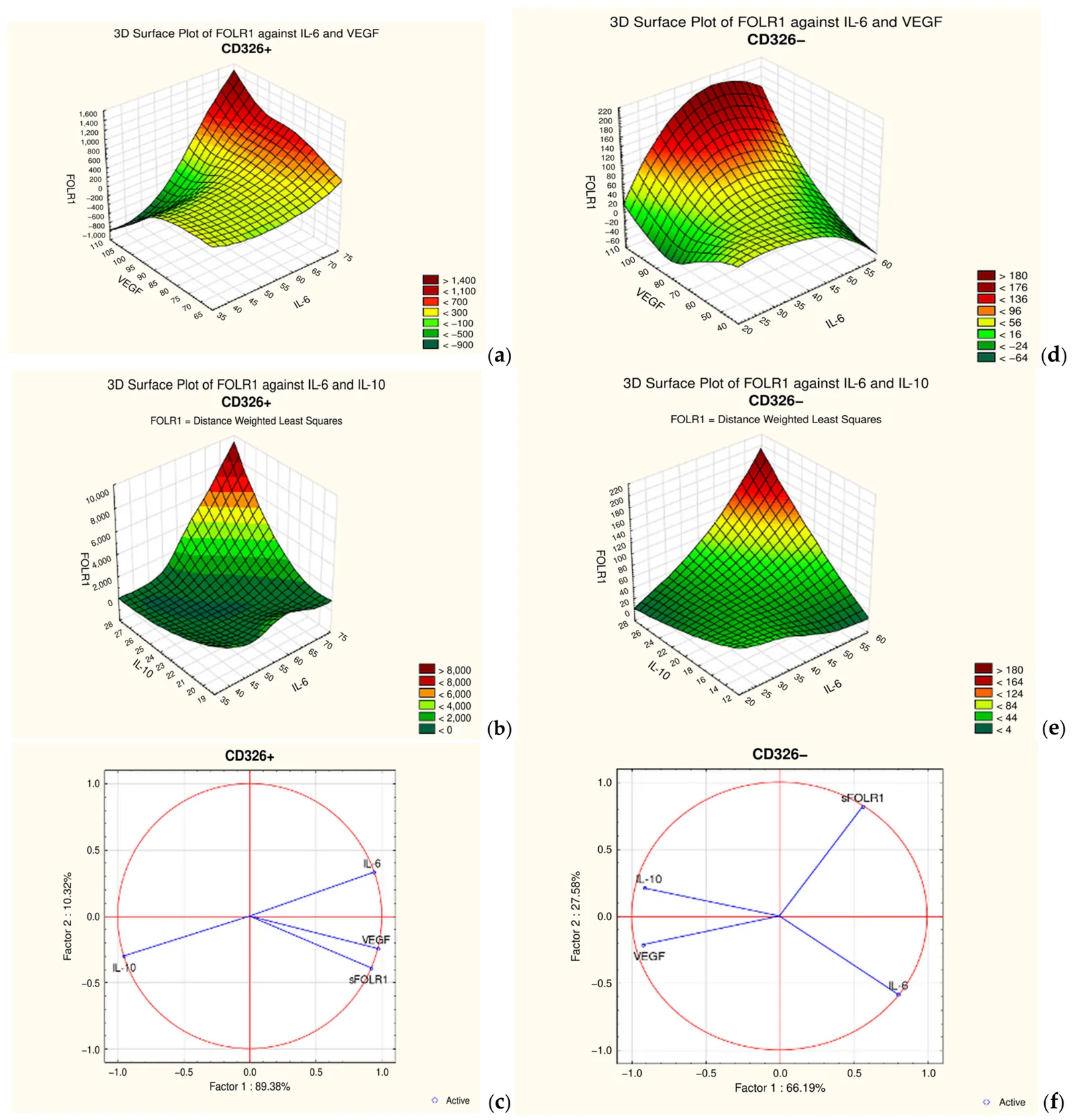
Relationships among sFOLR1, IL-6, IL-10, and VEGF in EpCAM/CD326-defined OVCAR-3 fractions. (**a**,**b**) Distance-weighted least-squares surfaces for CD326-positive fractions; (**d**,**e**) corresponding surfaces for CD326-negative fractions; (**c**,**f**) principal component loading plots for the positive and negative fractions.

The CD326-positive fraction showed a strong direct association between the time-dependent increases in sFOLR1 and VEGF (Pearson r = 0.9868, one-sided *p* = 0.0009) and between sFOLR1 and IL-6 (Spearman r = 1.000, one-sided *p* = 0.0083; Figure 4). The PCA loading plot provides a descriptive representation of these profiles (Figure 4c).

The CD326-negative fraction showed a divergent profile, with comparatively low sFOLR1 concentration. The surface models placed the joint high-cytokine region outside the experimentally observed sFOLR1 range (Figure 4d,e); these regions were not interpreted as evidence for biological associations. sFOLR1 production varies between CD326+ and CD326− fractions; the PCA loading plots (Figure 4c,f) of sFOLR1 indicate that the statistical association of highest VEGF, IL-6, and IL-10 would assume theoretical sFOLR1 concentrations below 200 pg/mg protein. Consistently, sFOLR1 did not cluster with the three cytokines in the PCA loading plot, and none were in the same quadrant as sFOLR1 (Figure 4f).

### 3.5. CD90-Defined Fractions: sFOLR1 and Cytokine Profiles in CD90-Positive and CD90-Negative

**Figure 5:**
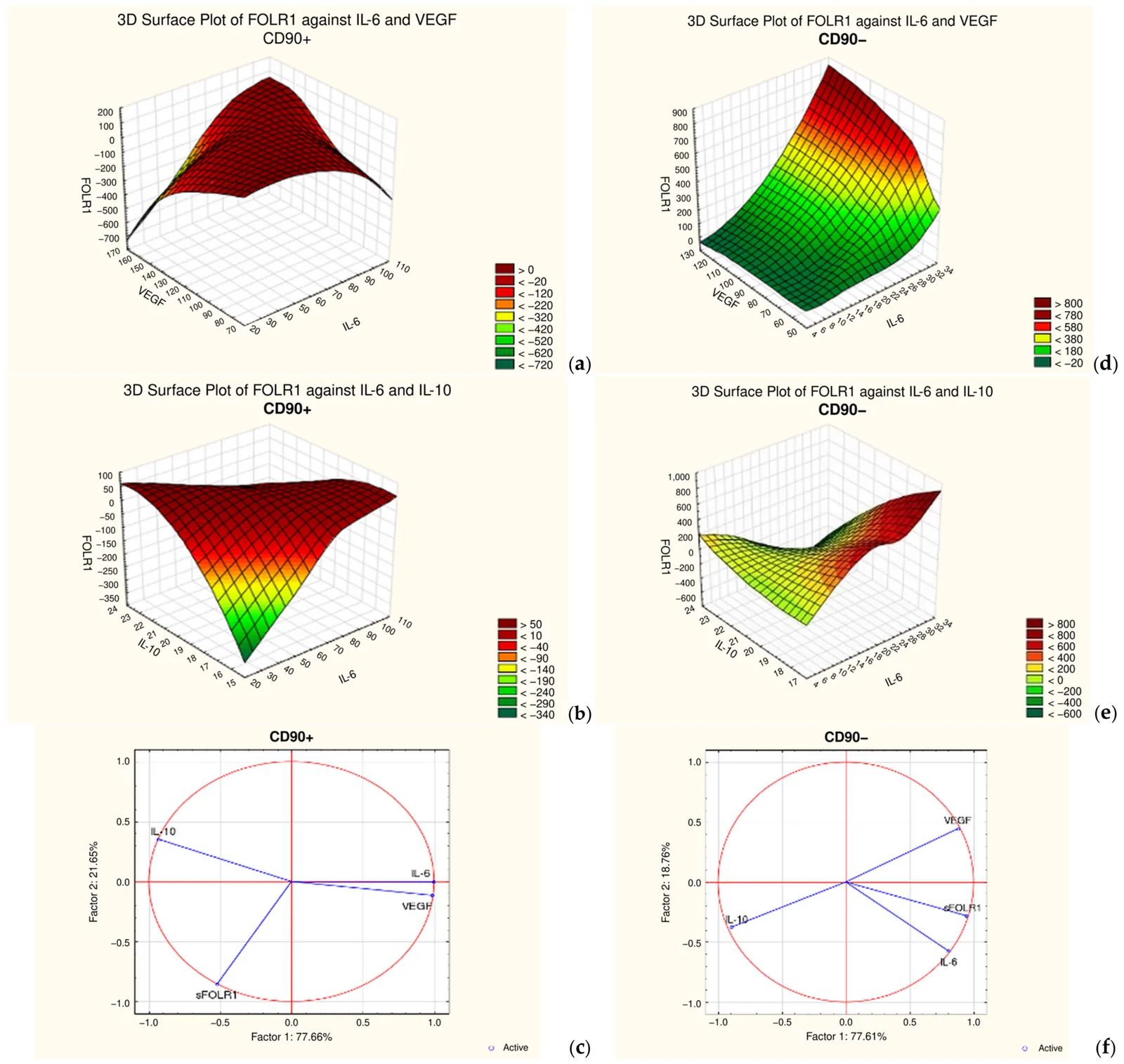
Secretome relationships in CD90-defined OVCAR-3 fractions. (**a**,**b**) Distance-weighted least-squares surfaces for CD90-positive fractions; (**d**,**e**) corresponding surfaces for CD90-negative fractions; (**c**,**f**) principal component loading plots for the two fractions.

In the CD90-positive OVCAR-3 fraction, the analysis did not identify an association between the sFOLR1 temporal profile and VEGF, IL-6, or IL-10 (Figure 5). VEGF and IL-6 were positively correlated (Pearson r = 0.9743, one-sided *p* = 0.0025). Their proximity in the PCA loading plot descriptively represents this pattern (Figure 5c), rather than an independent significance test. VEGF was inversely associated with IL-10 in both CD90-positive (Pearson r = −0.9493, one-tailed, *p* = 0.0068) and CD90-negative (Pearson r = −0.9127, one-sided *p* = 0.0153) fractions. The first PCA explained 77.66% of the variance in the CD90-enriched fraction and 77.61% in the CD90-depleted fraction (Figure 5c,f).

In the CD90-negative fraction, sFOLR1 and IL-6 were positively associated (Pearson r = 0.8918, one-tailed *p* = 0.0210). Modelled alignment of sFOLR1 with VEGF or IL-10 occurred only outside the measured sFOLR1 range.

### 3.6. ABCG2/CD338-Defined Fractions: sFOLR1 and Cytokine Profiles in ABCG2/CD338-Positive and ABCG2/CD338-Negative

**Figure 6:**
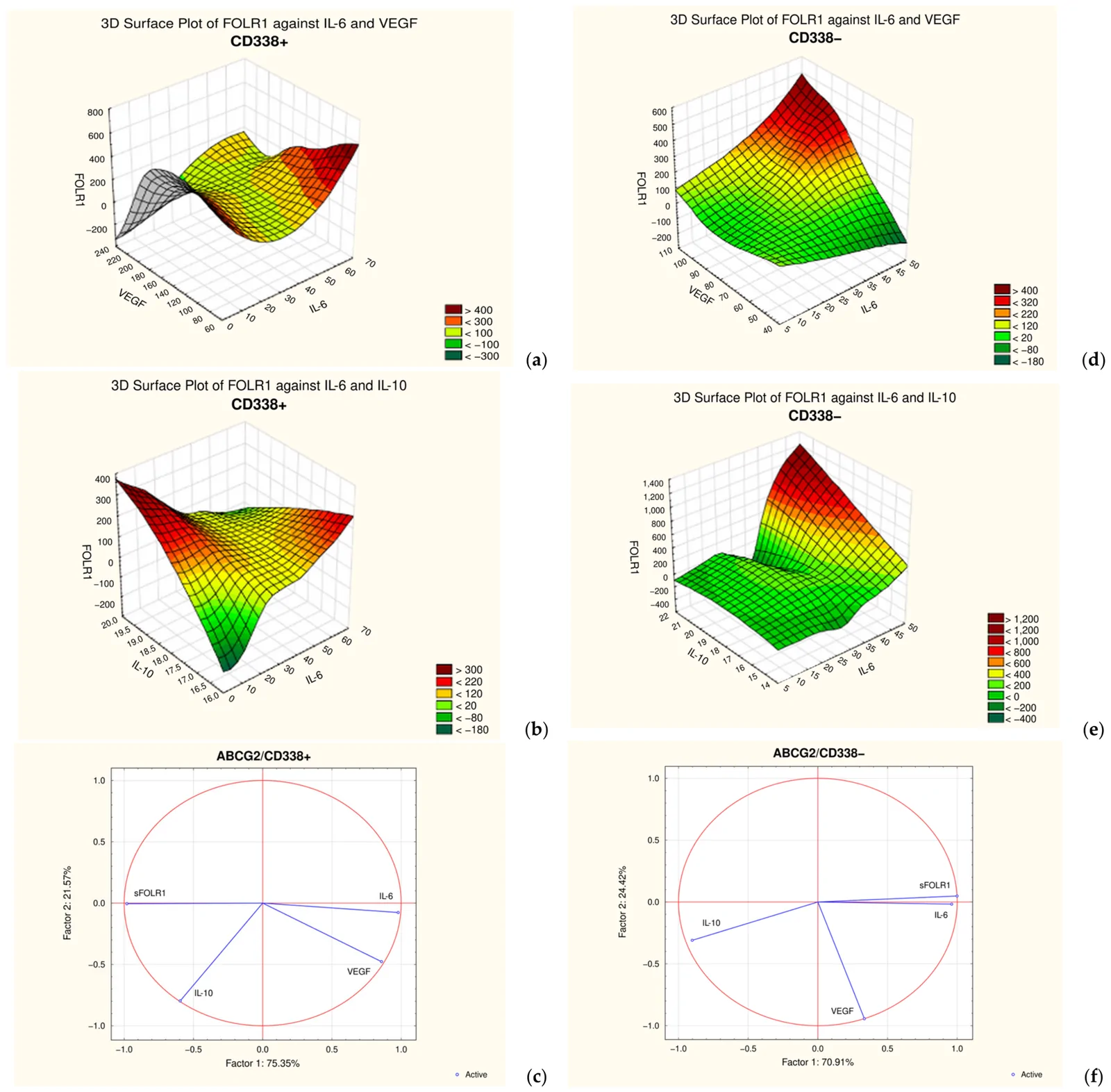
Relationships among sFOLR1, IL-6, IL-10, and VEGF in ABCG2/CD338-defined OVCAR-3 fractions. (**a**,**b**) Distance-weighted least-squares surfaces for CD338-positive fractions; (**d**,**e**) corresponding surfaces for CD338-negative fractions; (**c**,**f**) principal component loading plots for the positive and negative fractions, respectively.

ABCG2/CD338-positive fractions exhibited a high sFOLR1 concentration at 24 h followed by a progressive decline, whereas sFOLR1 increased in the corresponding depleted fractions (Figure 6). In the CD338-positive fraction, sFOLR1 declined alongside an overall increase in IL-6 and VEGF across the observation period. The principal component plot indicates alignment of sFOLR1/IL-10 and VEGF/IL-6 (Figure 6c).

In the CD338-negative OVCAR-3 subset, sFOLR1 was positively associated with IL-6 (Pearson r = 0.9639, one-sided *p* = 0.0041, Figure 6d,f) and inversely associated with IL-10 (Pearson r = −0.9001, *p* = 0.0187, Figure 6e). The first principal component accounted for 70.91% of the variation (Figure 6f), while the second component accounted for 24.42%. These analyses concern extracellular protein profiles and do not establish sensitivity to platinum-based treatment.

### 3.7. Pooled Comparison of Marker-Positive and Marker-Negative Fractions: Associations Between sFOLR1 Production and Cytokines Across the Marker-Enriched and Corresponding Depleted Cellular Fractions

**Figure 7:**
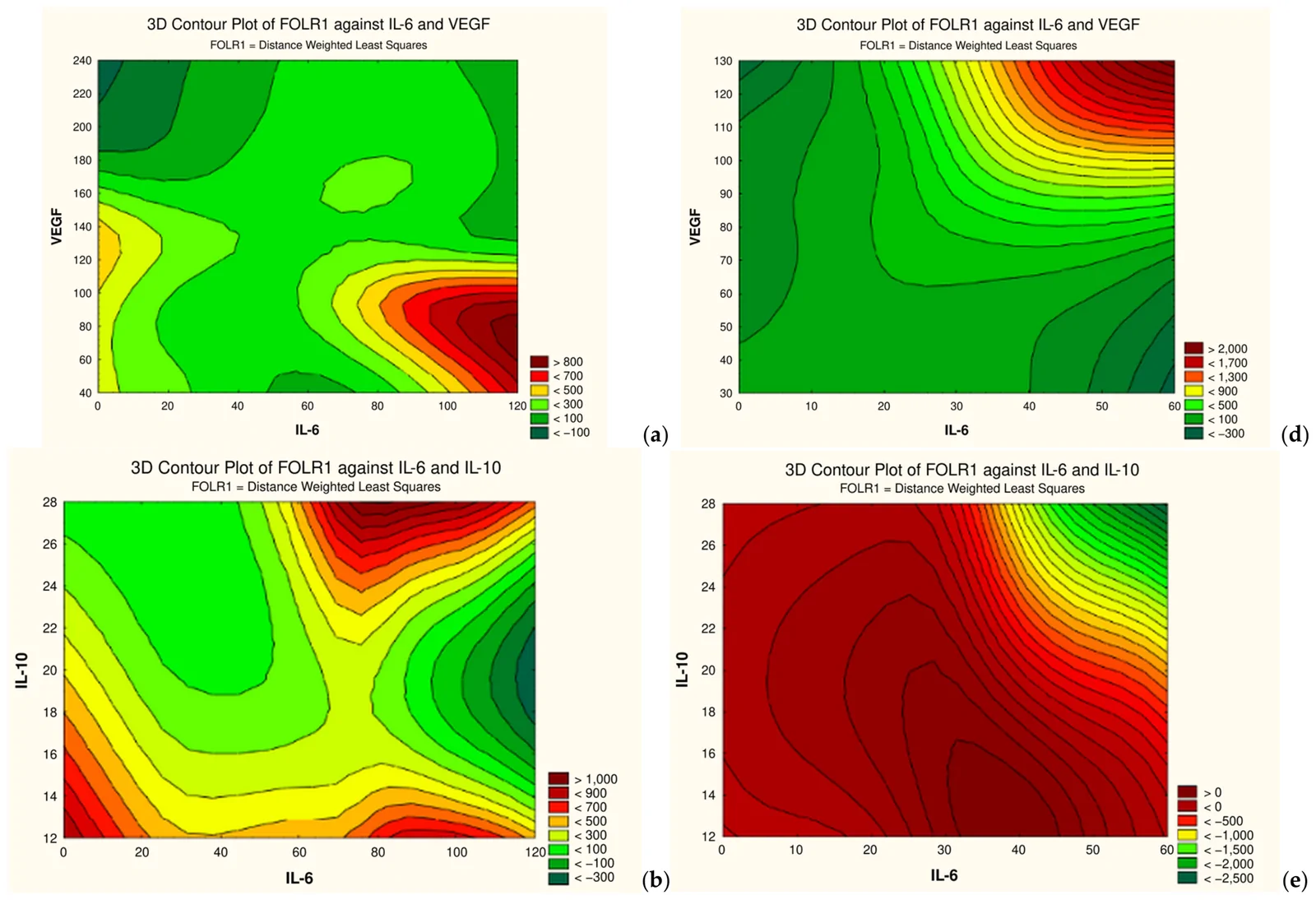

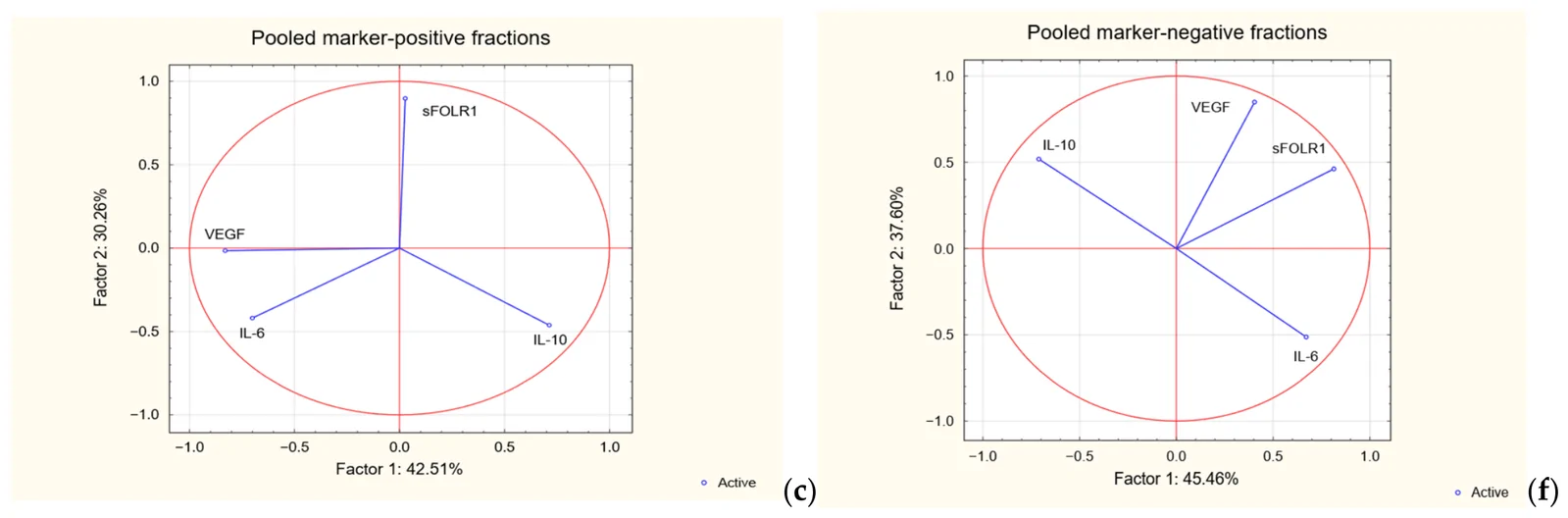
Pooled exploratory analysis of sFOLR1 and cytokine profiles. (**a**,**b**) Contour plots and (**c**) principal component loading plot for pooled marker-positive CD326+, CD133+, CD90+, and CD338+ fractions; (**d**,**e**) contour plots and (**f**) principal component loading plot for the corresponding pooled marker-negative fractions.

When all marker-positive fractions were pooled, sFOLR1 did not correlate with VEGF (Pearson r = −0.0350, two-sided *p* = 0.8835), IL-6 (r = −0.2064, *p* = 0.3826) or IL-10 (r = −0.2198, *p* = 0.3518). The first PCA yielded 42.51% of the variance in pooled marker-positive fraction, and 45.46% in marker-negative (Figure 7c,f). Relationships among VEGF, IL-6 and IL-10 were also explored using Pearson correlation and sFOLR1-IL-6-IL-10-VEGF contour plots (Figure 7a,b).

Across pooled marker-negative fractions, higher sFOLR1 was associated with higher VEGF. When enriched and depleted means were paired by marker and sampling time, VEGF (r = 0.5543, *p* = 0.0112) and IL-10 (r = 0.5244, *p* = 0.0176) were positively associated between groups; the IL-6 association did not meet the nominal significance threshold (r = 0.3604, *p* = 0.1186). The contour plots and PCA loading plot provide complementary descriptive visualizations of the pooled profiles (Figure 7d,f).

## 4. Discussion

The present analysis highlights that various OVCAR-3 cell subpopulations have distinct sFOLR1 and cytokine secretion profiles. The major finding is a set of phenotype-specific secretory patterns. CD133-positive and EpCAM/CD326-positive cells showed time-dependent sFOLR1 accumulation accompanied by VEGF and/or IL-6. CD90-positive cells displayed low sFOLR1 but exhibited coordinated VEGF and IL-6 values. ABCG2/CD338-positive cells showed a high early sFOLR1 value followed by progressive decline. These results describe heterogeneity within a common parental model and provide a rationale for evaluating extracellular sFOLR1 alongside marker-defined cellular context and cytokine profiles in subsequent studies.

The human ovarian epithelial cancer cell line OVCAR-3 (also known as NIH: OVCAR-3 or OVCAR3) is derived from a high-grade serous adenocarcinoma. As an epithelial cell line, OVCAR-3 overexpresses the EpCAM/CD326 protein and expresses the folate receptor alpha (FR-α or FOLR1) on the cell surface, and it showed one of the highest overlaps in the tumor antigen expression profile with the real-world tumor samples from ovarian cancer patients [39]. The presence of the membrane markers FOLR1, CD133, CD338, and CD90 on OVCAR-3 cells was quantified prior to the subset’s isolation to validate the data from the literature for the cell passages subjected to further testing. Within the displayed gated parental populations, flow cytometry showed 81.29% FOLR1-positive, 7.59% CD133-positive, 92.70% ABCG2/CD338-positive, and 2.58% CD90-positive events (Figure S1). The corresponding CD338 and CD90 percentages among all acquired events were 68.52% and 1.80%. These measurements neither demonstrate co-expression nor identify genetically distinct subsets, and they do not quantify post-isolation purity.

Clinically, circulating sFOLR1 has been associated with tumor burden, stage, outcome, and treatment response [13,15,40]. The present findings describe variation in extracellular sFOLR1 profiles among marker-defined fractions derived from a common parental OVCAR-3 model. Relative to 24 h, the reported temporal comparisons showed increased sFOLR1 in CD133-positive fractions at 72, 96, and 120 h, in CD133-negative fractions at all subsequent sampling times, and in CD338-negative fractions at 72, 96, and 120 h; the CD338-positive fraction instead showed a decline. No significant temporal change was reported for the CD90-positive and CD326-negative fractions, whereas CD90-negative fractions increased at 96 and 120 h and CD326-positive fractions at 72, 96, and 120 h. These marker-based comparisons do not establish reciprocal epithelial–mesenchymal differentiation or mutual exclusivity of CD90 and CD326 expression.

Each marker-defined OVCAR-3 fraction displayed a distinctive cytokine pattern, with several convergences or divergences among the studied proteins. Associations among the analytes were explored across the five time-point profiles within each fraction. In many cases, IL-6, IL-10, or VEGF levels rose or fell significantly. The CD326-positive profile is consistent with the epithelial distribution of FOLR1 and with the high prevalence of FOLR1 expression in high-grade serous ovarian carcinoma [19]. Its close alignment with VEGF and IL-6 suggests that sFOLR1 release may occur within a broader epithelial secretory program that includes angiogenic and inflammatory signals. CD133-positive fractions showed no corresponding relationship with IL-10 but showed a correlation between sFOLR1 and VEGF and IL-6. CD90-positive fractions showed a strong correlation between VEGF and IL-6, but little change in sFOLR1. Thus, the cytokine and soluble-receptor profiles did not vary uniformly within this fraction. Conversely, ABCG2/CD338-positive fractions began with high sFOLR1 and then declined while IL-6 and VEGF increased. These findings describe variation in sFOLR1 and cytokine profiles among marker-defined fractions. sFOLR1 may instead reflect transient shedding, cell adaptation, altered receptor turnover, or changes in viable cell numbers; none of these mechanisms was directly tested here. In the corresponding CD338-negative fraction, increasing sFOLR1 accompanied decreasing IL-10 concentrations. These opposing temporal profiles indicate that the two extracellular analytes did not vary in parallel; they do not establish a direct regulatory relationship.

VEGF concentrations increased relative to 24 h at 72, 96 and 120 h in the CD133-positive, CD338-positive, and CD90-positive fractions, whereas the CD326-negative fraction decreased at 72 and 96 h. Epithelial–mesenchymal transition and drug resistance have been discussed in high-grade serous ovarian cancer [41], but these functional properties were not assessed in the present study.

These observations provide a rationale for testing whether joint measurement of sFOLR1, IL-6, VEGF, and tumor phenotype adds information in independent models and clinical cohorts; no diagnostic or prognostic performance, or superiority over an individual marker, was evaluated here. Circulating sFOLR1 has previously been investigated for ovarian cancer diagnosis and surveillance [40], while additional markers may enrich and refine findings.

### Limitations

Only one established ovarian cell line, OVCAR-3, was studied, and immunomagnetic enrichment yielded marker-enriched populations rather than clonally pure ones. This pilot study was designed to characterize variation in sFOLR1 and cytokine profiles among marker-enriched and corresponding depleted fractions within the OVCAR-3 model. The use of a common parental line allowed comparisons within the same cellular background, consistent with the exploratory objective. The study was not designed to establish reproducibility across ovarian cancer models; the observed profiles may therefore reflect OVCAR-3-specific biology. Independent validation in additional cell lines is required to assess their generalizability. Candidate models for future validation include SKOV-3 and A2780cis; establish marker representation and drug-response phenotypes in each model before comparing the corresponding fractions. The same marker-defined populations cannot be assumed to occur at comparable frequencies across ovarian cancer models. Broader validation should include established and patient-derived models representing primary tumors and metastatic disease, with at least two models from each origin and functional evaluation of cancer stem-like and drug-resistant phenotypes. Immunomagnetic enrichment was not followed by flow-cytometric purity assessment. Marker expression alone cannot establish functional stemness or drug resistance. The resazurin assay assessed metabolic activity without drug exposure. Drug-response assays and complementary assessments of self-renewal and tumor-initiating capacity are required to determine the functional properties of these fractions.

Immunomagnetic separation generated fractions enriched for CD133, EpCAM/CD326, and ABCG2/CD338, together with the corresponding depleted fractions, from the parental OVCAR-3 culture. CD90 enrichment was performed after indirect co-culture with primary mesenchymal stromal cells using a protocol adapted from Yang et al. [35]. The resulting fractions were not independently established cell lines. The distance-weighted surface and contour plots are fitted visual aids, and several plotted axes extend beyond measured values. Their geometry depends on the fitting choices and the small number of time-point means; therefore, it should not be interpreted as a mechanistic model. The experiments were not cross-validated in a second ovarian cancer cell line.

The temporal comparisons describe extracellular protein profiles from 24 to 120 h, using the 24 h culture measurement as the reference. The 0 h samples were blank controls collected immediately after cell resuspension in culture medium. They characterize the initial extracellular background but do not provide paired cellular FOLR1 measurements or resolve changes occurring between resuspension and the first 24 h collection. The onset and mechanism of FOLR1 release, therefore, remain undetermined.

Cell-surface FOLR1 was assessed in the parental culture, whereas soluble FOLR1 was measured in the supernatants of the isolated fractions. The absence of paired cellular and extracellular measurements prevents attribution of the soluble profiles to differences in receptor abundance, release, turnover, or viable cell number. The processing protocol combined normalization to total supernatant protein with adjustment to 10^6^ counted cells. These adjusted measurements do not establish a secretion rate per viable cell or exclude contributions from differences in cell recovery and metabolic state. In the parental OVCAR-3 culture, 2.58% of events were CD90-positive within the displayed gated population; therefore, stromal co-culture was used before immunomagnetic enrichment of the CD90-positive fraction. This preceding co-culture introduces a potential source of variation independent of CD90 expression. Matched conditioning controls and post-harvest identity and purity assessments could help distinguish these effects.

These limitations render the results exploratory but provide a sound rationale for targeted validation.

## 5. Conclusions

Distinct extracellular sFOLR1 and cytokine secretion profiles were highlighted across marker-enriched and corresponding depleted OVCAR-3 fractions. CD326-positive and CD133-positive fractions showed coordinated sFOLR1, VEGF, and IL-6 patterns, while CD90-positive and ABCG2/CD338-positive fractions exhibited dissociation between sFOLR1 and cytokine concentrations. These findings describe marker-associated secretory heterogeneity within an OVCAR-3 population and derived fractions. Integrating sFOLR1 with phenotype-specific cytokine signals may improve biological interpretation and could generate composite biomarker strategies that include cytokines. Further validation in independent and patient-derived models is required.

## Data Availability

The data supporting the findings are contained within the article and Supplementary Materials; raw data are available from the corresponding author upon reasonable request.

## Supplementary Materials

The following supporting information can be downloaded at: https://www.mdpi.com/article/10.3390/biom16101462/s1, Figure S1. Assessment of membrane marker expression in OVCAR-3 by flow cytometry; a. Unstained OVCAR-3, without PE detection; b. Expression of folate receptor FR-α or FOLR1 on the cell membrane, quantified as 81.29% within the displayed gated population, evidenced by PE-labelled anti-FR-α antibody; c. Expression of Prominin-1/CD133 (7.59%), measured by labelling with PE-anti-CD133 antibody; d. Expression of ABCG2/CD338 (92.70%), evidenced by PE-anti-CD338 antibody; e. Cells without PerCP/Cyanine5.5-labelled anti-CD90 antibody staining; f. Expression of Thy-1 or CD90 in OVCAR-3 (2.58%), staining with PerCP-Cyanine5.5-labelled anti-CD90 antibody. Figure S2. Morphology of corresponding marker-positive and marker-negative cell fractions after 72 h of culture; inverted microscope images (acquired with Observer D1 microscope from Zeiss, Oberkochen, Germany): (a) CD133+ (left) and CD133− (right), in white light, 10× objective; (b) CD90+ vs. CD90− in white light, 10× objective); (c) CD326+ (left) versus CD326− cells (right, 20× objective); (d) CD338+ (left) versus CD338− cells (right, 40× objective). Figure S3. Time-dependent metabolic activity of marker-enriched OVCAR-3 fractions after 24, 48, 72, 96, and 120 h of culture in 24-well plates. The y-axis shows fluorescence intensity following incubation with resazurin dye (Alamar Blue, Invitrogen, Carlsbad, CA, USA), with emission measured at 620 nm. Fluorescence was used as an indicator of viable-cell metabolic activity and does not directly establish proliferation rates. Figure S4. (a–d) Quantitative evaluation of soluble folate receptor (FOLR1) concentrations in marker-enriched fractions and corresponding depleted OVCAR-3 fractions. Figure S5. (a–d) Quantitative in vitro evaluation of VEGF-A concentrations in marker-enriched and corresponding depleted OVCAR-3 fractions. Figure S6. (a–d) Quantitative in vitro evaluation of Interleukin-6 concentrations in marker-enriched and corresponding depleted OVCAR-3 fractions. Figure S7. (a–d) Quantitative evaluation of Interleukin-10 concentrations in marker-enriched and corresponding depleted fractions derived from the parental OVCAR-3 culture. Table S1. Statistical significance of the variations in VEGF concentration (pg/mg protein) at 72, 96 and 120 h relative to the 24 h reference concentration, in marker-enriched and corresponding depleted OVCAR-3 fractions. Table S2. Statistical significance of the variations in IL-6 concentration at 48, 72, 96 and 120 h, relative to the 24-hours reference concentration, in marker-enriched and corresponding depleted OVCAR-3 fractions. Table S3. Statistical significance of the variations in IL-10 concentration at 48, 72, 96 and 120 h, relative to the 24-hours reference concentration, in marker-enriched and corresponding depleted OVCAR-3 fractions.

## Abbreviations

The following abbreviations are used in this manuscript:

FRα	Folate Receptor Alpha
FOLR1	Folate Receptor 1
sFOLR1	Soluble Folate Receptor 1
PARPi	Poly-ADP-ribose polymerase inhibitors
CA-125	Cancer Antigen 125
DNA	Deoxyribonucleic acid
CAR-T	Chimeric antigen receptor T
VEGF	Vascular endothelial growth factor
IL-6	Interleukin-6
IL-10	Interleukin-10
VEGF-A	Vascular endothelial growth factor A
