# Supplementary material for: Distinct sFOLR1 and Cytokine Secretion Profiles Across Marker-Enriched OVCAR-3 Ovarian Cancer Cell Subpopulations: A Pilot Study
Source: Biomolecules. 2026 Oct 7;16(10):1462. doi: 10.3390/biom16101462 (PMC13702145; doi:10.3390/biom16101462)
Supplement: Supplementary file 1 [file biomolecules-16-01462-s001.zip › biomolecules-4548607-supplementary.pdf]

Supplementary material

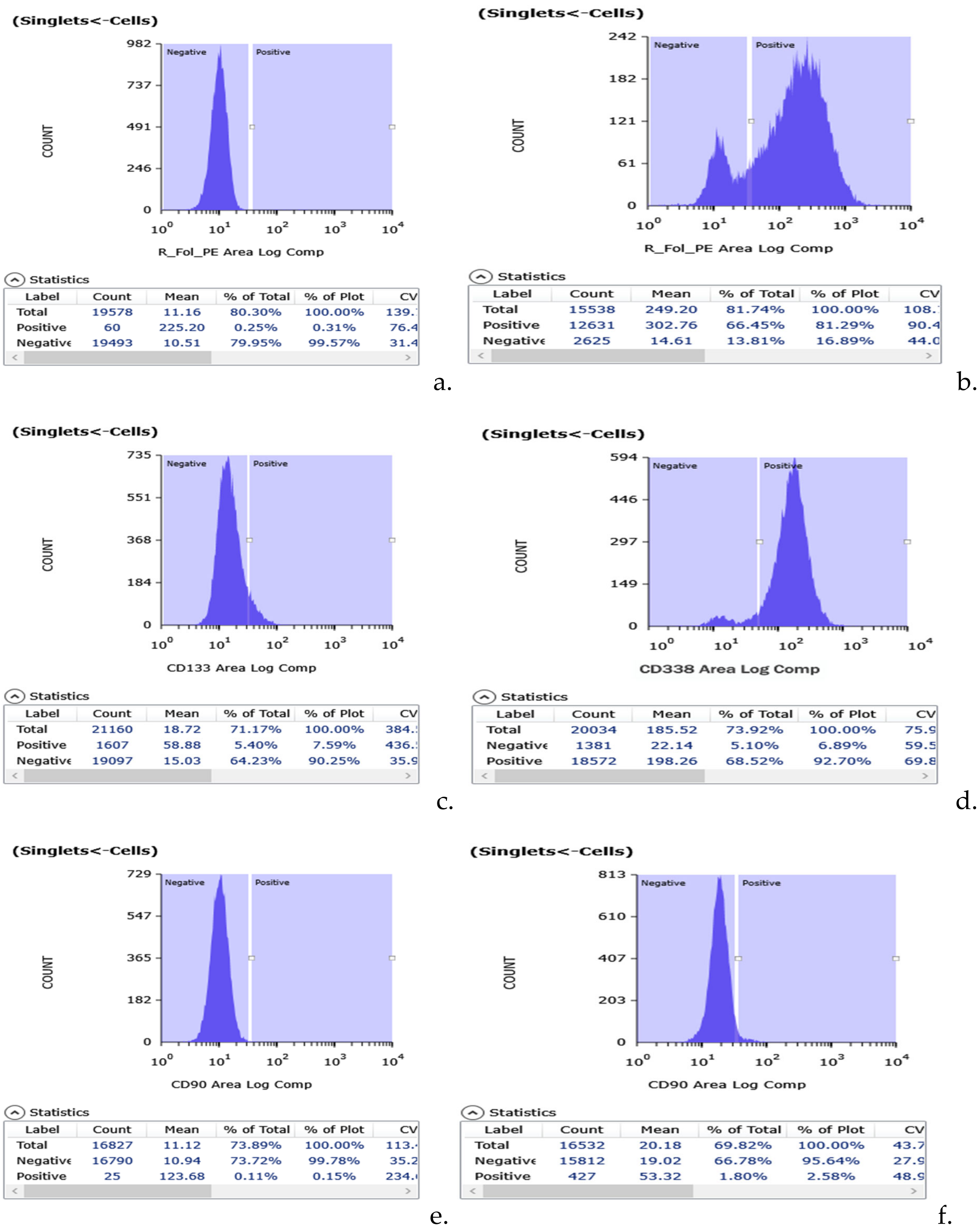

Figure S1. Assessment of membrane marker expression in OVCAR-3 by flowcytometry; a. Unstained OVCAR-3, without PE detection; b. Expression of folate receptor FR-α or FOLR1 on the cell membrane, quantified as 81.29% within the displayed gated population, evidenced by PE-labelled anti-FR-α antibody; c. Expression of Prominin-1/CD133 (7.59%), measured by labelling with PE-anti-

CD133 antibody; d. Expression of ABCG2/CD338 (92.70%), evidenced by PE-anti-CD338 antibody; e. Cells without PerCP-Cyanine5.5-labelled anti-CD90 antibody staining; f. Expression of Thy-1 or CD90 in OVCAR-3 (2.58%), staining with PerCP-Cyanine5.5-labelled anti-CD90 antibody.

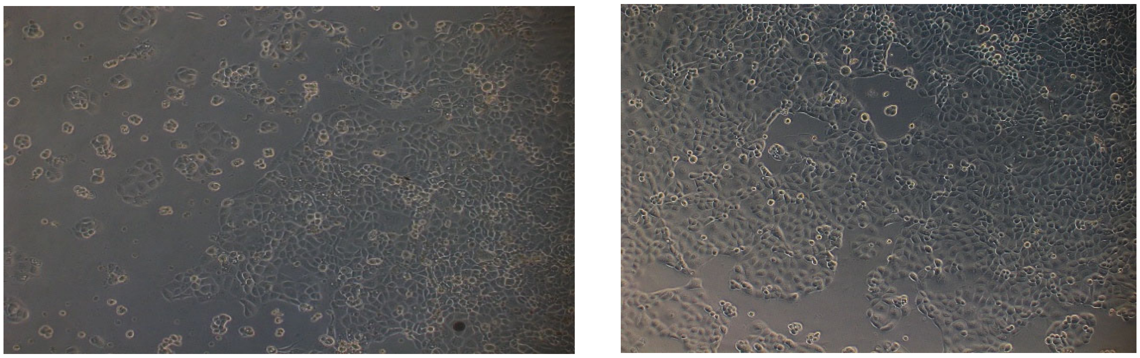

a.

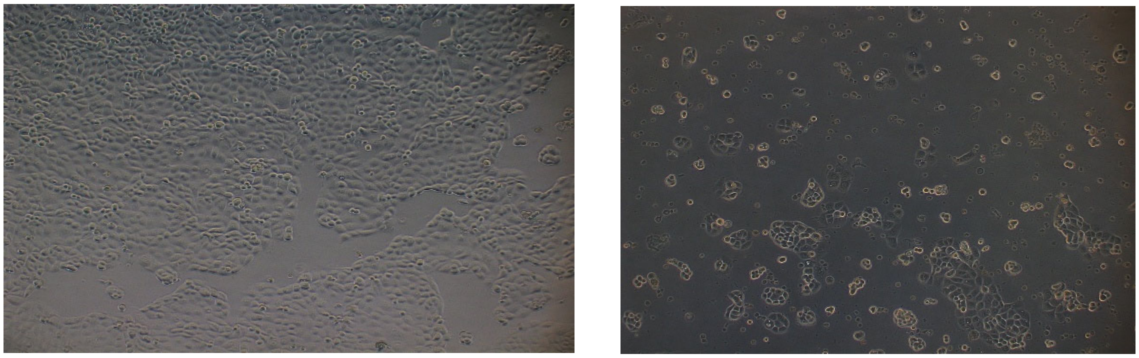

b.

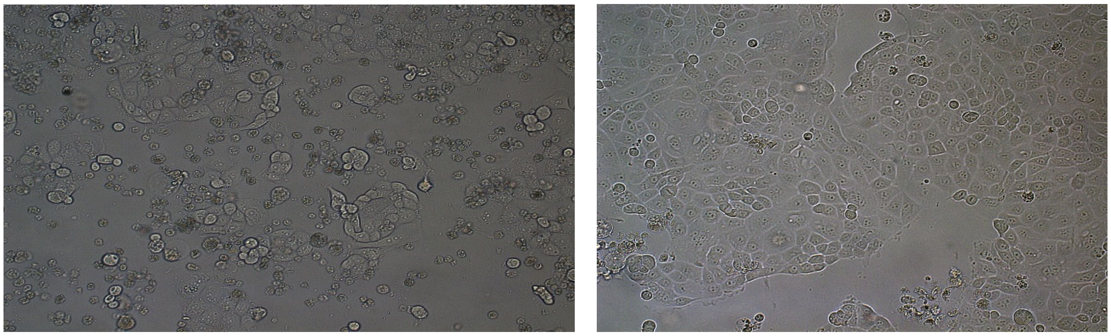

c.

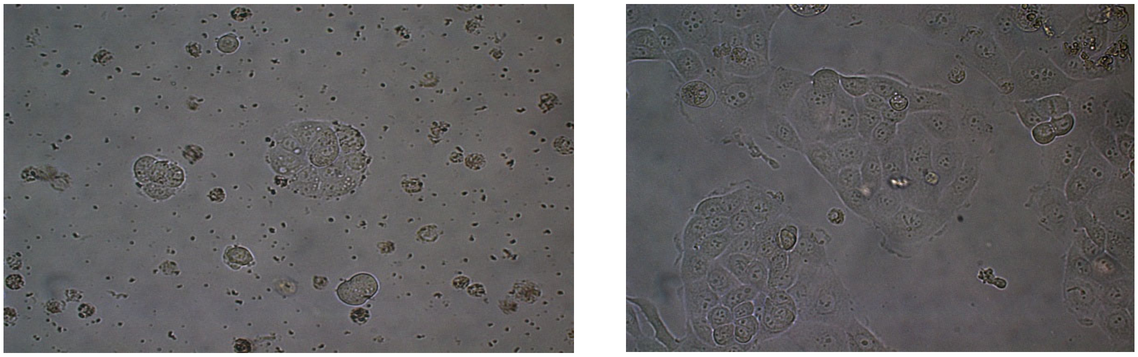

d.

Figure S2. Morphology of corresponding marker-positive and marker-negative cell fraction after 72 h of cultivation; inverted microscope images (acquired with Observer D1 microscope from Zeiss, Oberkochen, Germany): (a) CD133+ (left) and CD133− (right), in white light, 10x objective; (b) CD90+ vs CD90− in white light, 10x objective; (c) CD326+ (left) versus CD326− cells (right, 20x objective); d.) CD338+ (left) versus CD338− cells (right, 40x objective). The + and − indicate marker-positive and marker-negative fractions, respectively.

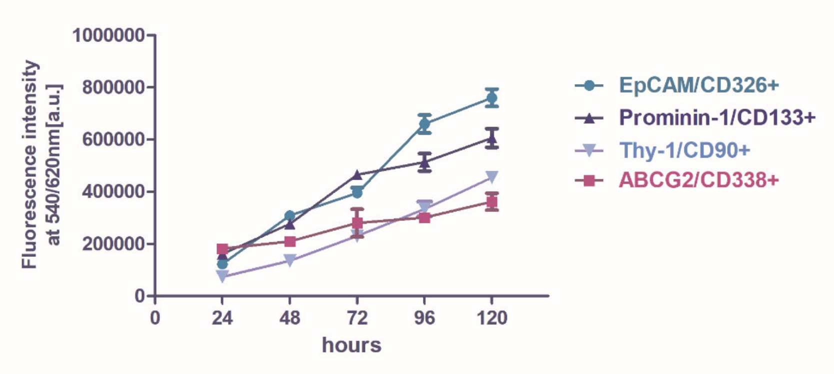

Figure S3. Time-dependent metabolic activity of marker-enriched OVCAR-3 fractions after 24, 48, 72, 96, and 120 h of culture in 24-well plates. The y-axis shows fluorescence intensity following incubation with resazurin dye (Alamar Blue, Invitrogen, Carlsbad, CA, USA), with emission measured at 620 nm. Fluorescence was used as an indicator of viable-cell metabolic activity and does not directly establish proliferation rates.

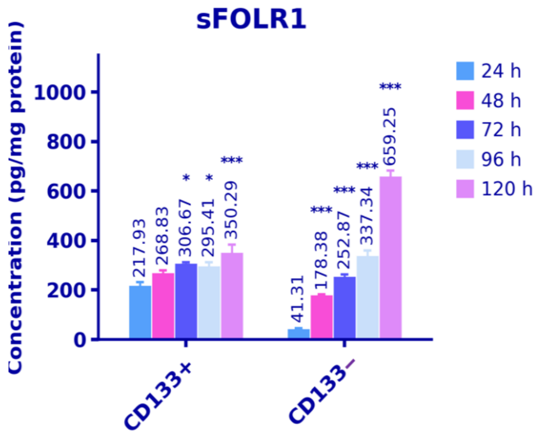

a.

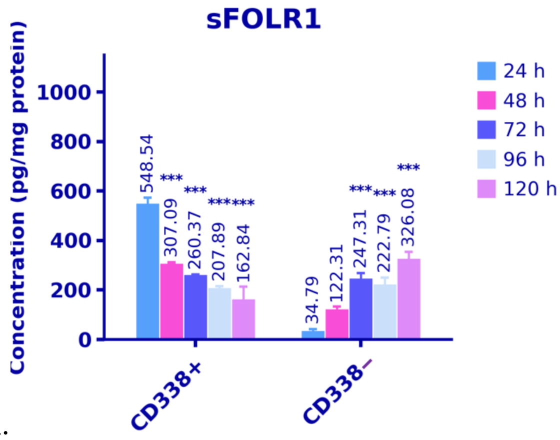

b.

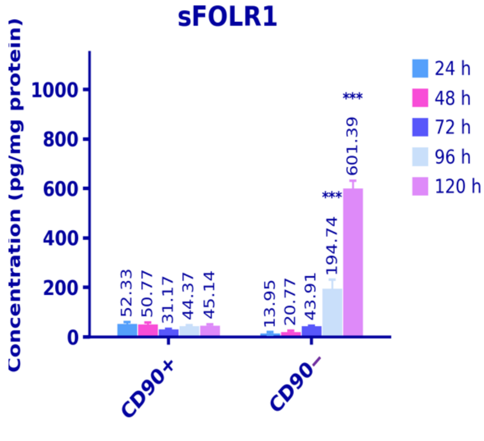

c.

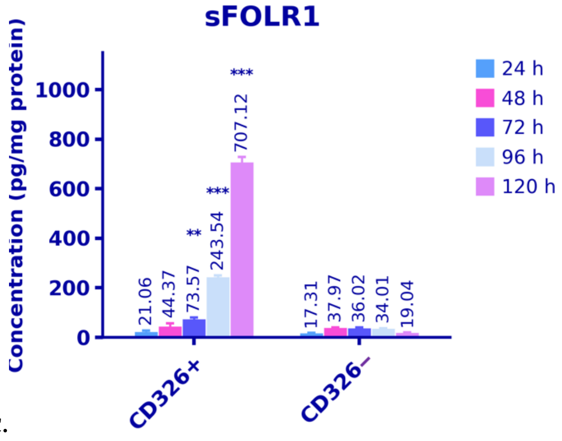

d.

Figure S4. (a–d) Quantitative evaluation of soluble folate receptor (sFOLR1) concentrations in marker-enriched fractions and corresponding depleted OVCAR-3 fractions. Asterisks denote statistical significance: * $p < 0.05$, ** $p < 0.01$. and $p < 0.001$.

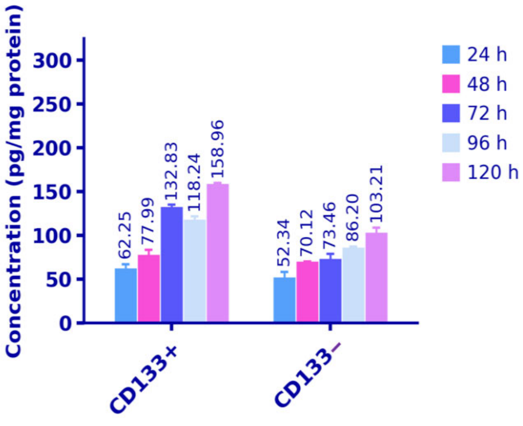

a.

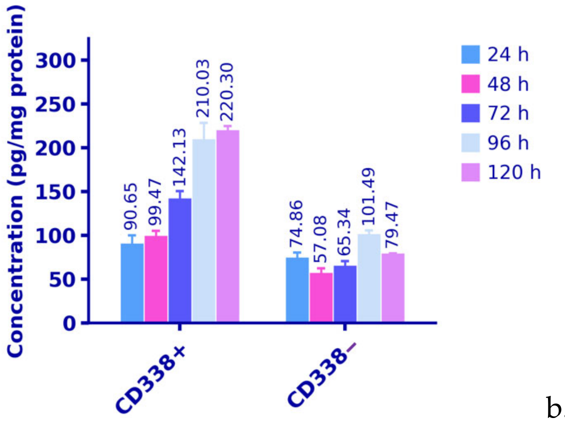

b.

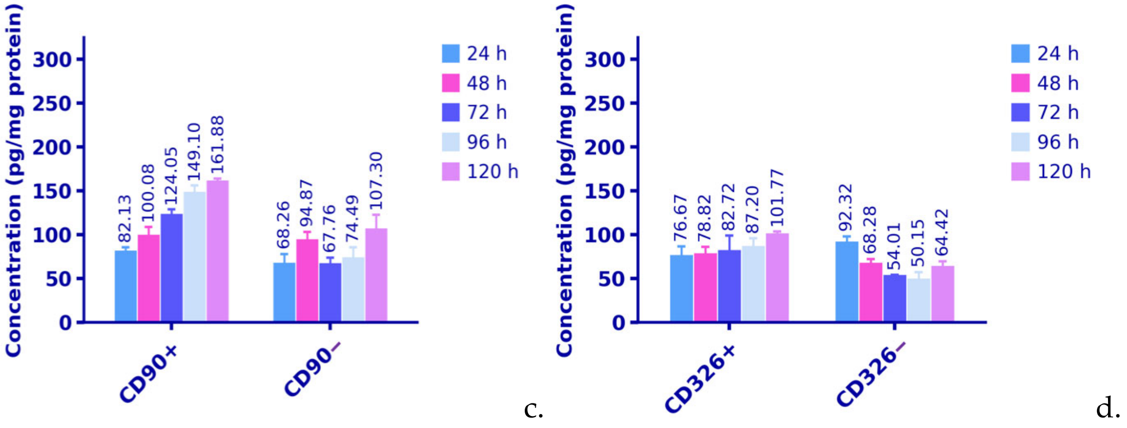

Figure S5. (a–d) Quantitative *in vitro* evaluation of VEGF-A concentrations in marker-enriched and corresponding depleted OVCAR-3 fractions.

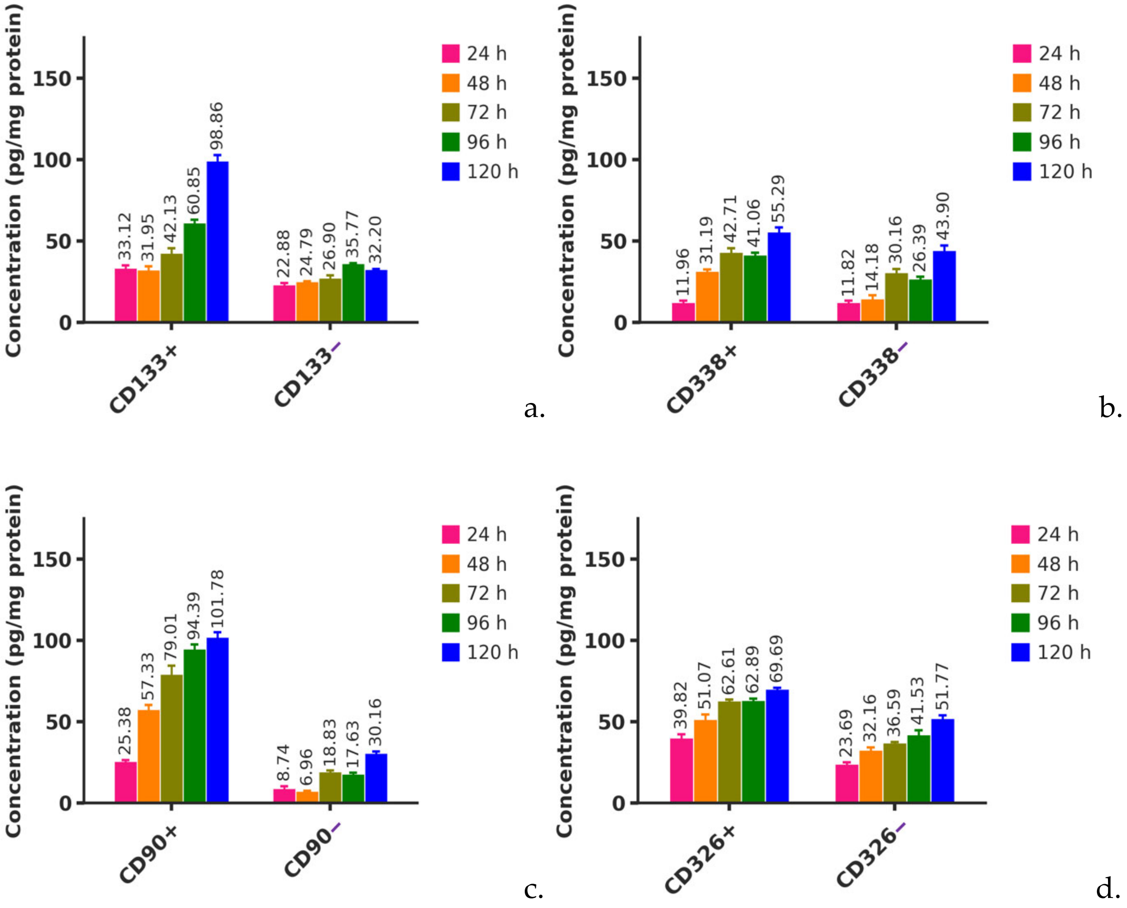

Figure S6. (a–d) Quantitative *in vitro* evaluation of Interleukin-6 concentrations in marker-enriched and corresponding depleted OVCAR-3 fractions.

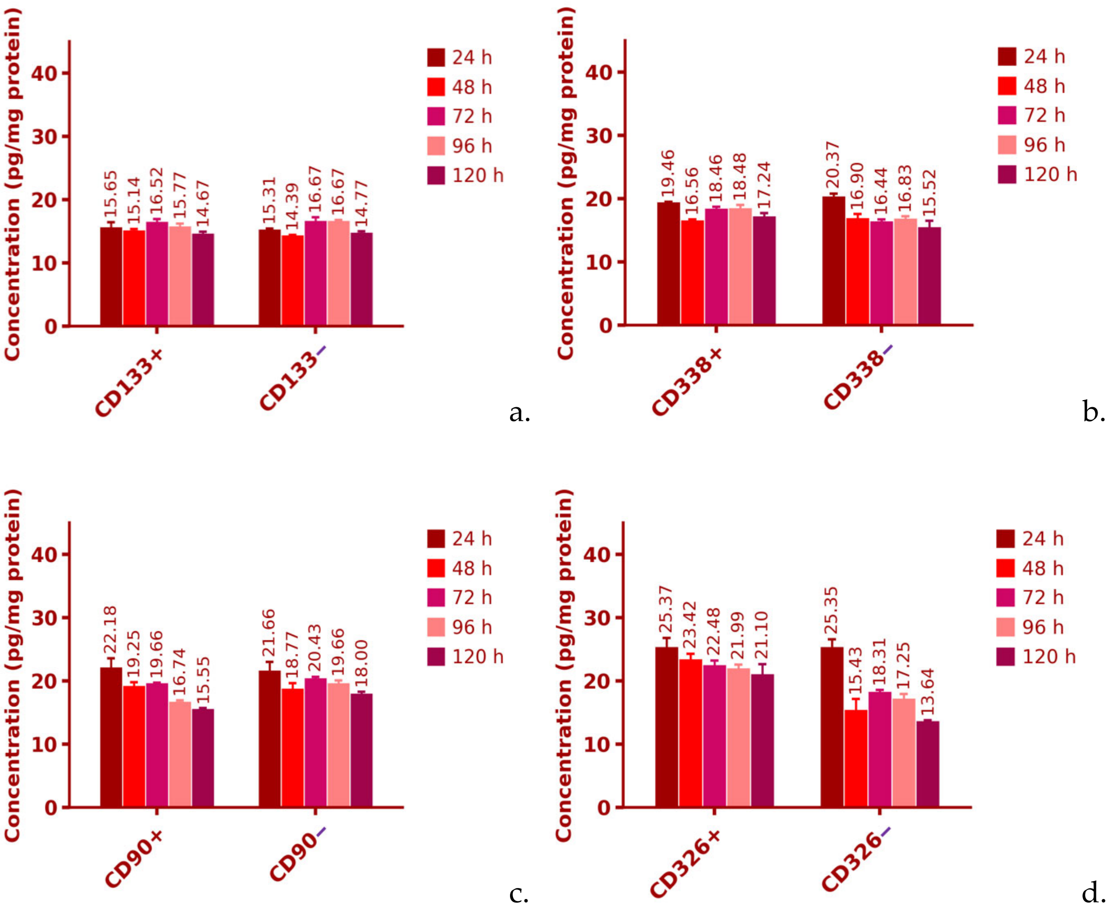

Figure S7. (a–d) Quantitative evaluation of Interleukin-10 concentrations in marker-enriched and corresponding depleted fractions derived from the parental OVCAR-3 culture.

Table S1. Statistical significance of the variations in VEGF concentration (pg/mg protein) at 72, 96 and 120 h, relative to the 24 h reference concentration, in marker-enriched and corresponding depleted OVCAR-3 fractions. Significance: * $p < 0.05$, ** $p < 0.01$. and $p < 0.001$. The + and – indicate marker-positive and marker-negative fractions, respectively.

**Two-way repeated-measure ANOVA VEGF**				
**24 hours vs 72 hours**				
Row Factor	Difference	t ratio	*p* value	Summary
CD133+	70.58	6.649	$p < 0.001$	***
CD338+	51.48	4.850	$p < 0.001$	***
CD90+	41.92	3.949	$p < 0.01$	**
CD326–	−38.32	3.610	$p < 0.01$	**
**24 hours vs 96 hours**				
Row Factor	Difference	t ratio	p value	Summary
CD133+	55.99	5.275	$p < 0.001$	***

CD133–	33.86	3.189	$p < 0.05$	*
CD338+	119.4	11.25	$p < 0.001$	***
CD90+	66.97	6.309	$p < 0.001$	***
CD326–	−42.17	3.973	$p < 0.01$	**
**24 hours vs 120 hours**				
Row Factor	Difference	t ratio	*p* value	Summary
CD133+	96.71	9.111	$p < 0.001$	***
CD133–	50.86	4.792	$p < 0.001$	***
CD338+	129.7	12.21	$p < 0.001$	***
CD90+	79.75	7.513	$p < 0.001$	***
CD90–	39.04	3.678	$p < 0.01$	**

Table S2. Statistical significance of the variations in IL-6 concentration at 48, 72, 96 and 120 h, relative to the 24 h reference concentration, in marker-enriched and corresponding depleted OVCAR-3 fractions. Significance: * $p < 0.05$, ** $p < 0.01$. and $p < 0.001$. The + and − indicate marker-positive and marker-negative fractions, respectively.

**Two-way repeated-measure ANOVA IL-6**				
**24 vs 48 hours**				
Row Factor	Difference	t ratio	*p* value	Summary
CD338+	19.23	5.653	$p < 0.001$	***
CD90+	31.96	9.394	$p < 0.001$	***
CD326+	11.25	3.306	$p < 0.05$	*
**24 vs 72 hours**				
Row Factor	Difference	t ratio	*p* value	Summary
CD338+	30.75	9.040	$p < 0.001$	***
CD338–	18.34	5.393	$p < 0.001$	***
CD90+	53.63	15.77	$p < 0.001$	***
CD90–	10.09	2.968	$p < 0.05$	*
CD326+	22.79	6.699	$p < 0.001$	***
CD326–	12.90	3.791	$p < 0.01$	**
**24 vs 96 hours**				
Row Factor	Difference	t ratio	*p* value	Summary
CD133+	27.73	8.153	$p < 0.001$	***
CD133–	12.89	3.790	$p < 0.01$	**
CD338+	29.10	8.555	$p < 0.001$	***
CD338–	14.57	4.284	$p < 0.01$	**
CD90+	69.01	20.29	$p < 0.001$	***
CD326+	23.07	6.783	$p < 0.001$	***
CD326–	17.84	5.245	$p < 0.001$	***
**24 vs 120 hours**				
Row Factor	Difference	t ratio	*p* value	Summary
CD133+	65.74	19.33	$p < 0.001$	***
CD338+	43.33	12.74	$p < 0.001$	***
CD338–	32.08	9.431	$p < 0.001$	***
CD90+	76.40	22.46	$p < 0.001$	***
CD90–	21.42	6.298	$p < 0.001$	***

CD326+	29.86	8.779	$p < 0.001$	***
CD326–	28.08	8.254	$p < 0.001$	***

Table S3. Statistical significance of the variations in IL-10 concentration at 48, 72, 96 and 120 h, relative to the 24 h reference concentration, in marker-enriched and corresponding depleted OVCAR-3 fractions. Significance: * $p < 0.05$, ** $p < 0.01$. and $p < 0.001$. The + and – indicate marker-positive and marker-negative fractions, respectively.

**Two-way repeated-measure ANOVA IL-10**				
**24 vs 48 hours**				
Row Factor	Difference	t ratio	*p* value	Summary
CD338+	−2.908	3.637	$p < 0.01$	**
CD338–	−3.475	4.347	$p < 0.01$	**
CD90+	−2.929	3.664	$p < 0.01$	**
CD90–	−2.886	3.610	$p < 0.01$	**
CD326–	−9.921	12.41	$p < 0.001$	***
**24 vs 72 hours**				
Row Factor	Difference	t ratio	*p* value	Summary
CD338–	−3.930	4.915	$p < 0.001$	***
CD90+	−2.516	3.147	$p < 0.05$	*
CD326+	−2.894	3.619	$p < 0.01$	**
CD326–	−7.045	8.810	$p < 0.001$	***
**24 vs 96 hours**				
Row Factor	Difference	t ratio	*p* value	Summary
CD338–	−3.546	4.434	$p < 0.001$	***
CD90+	−5.439	6.802	$p < 0.001$	***
CD326+	−3.385	4.234	$p < 0.01$	**
CD326–	−8.103	10.13	$p < 0.001$	***
**24 vs 120 hours**				
Row Factor	Difference	t ratio	*p* value	Summary
CD338–	−4.852	6.068	$p < 0.001$	***
CD90+	−6.628	8.289	$p < 0.001$	***
CD90–	−3.662	4.579	$p < 0.001$	***
CD326+	−4.276	5.347	$p < 0.001$	***
CD326–	−11.72	14.66	$p < 0.001$	***
